# Impact of Water Chemistry, Pipe Material and Stagnation on the Building Plumbing Microbiome

**DOI:** 10.1371/journal.pone.0141087

**Published:** 2015-10-23

**Authors:** Pan Ji, Jeffrey Parks, Marc A. Edwards, Amy Pruden

**Affiliations:** Via Department of Civil and Environmental Engineering, Virginia Tech, Blacksburg, Virginia, United States of America; U. S. Salinity Lab, UNITED STATES

## Abstract

A unique microbiome establishes in the portion of the potable water distribution system within homes and other buildings (i.e., building plumbing). To examine its composition and the factors that shape it, standardized cold water plumbing rigs were deployed at the treatment plant and in the distribution system of five water utilities across the U.S. Three pipe materials (copper with lead solder, CPVC with brass fittings or copper/lead combined pipe) were compared, with 8 hour flush cycles of 10 minutes to simulate typical daily use patterns. High throughput Illumina sequencing of 16S rRNA gene amplicons was employed to profile and compare the resident bulk water bacteria and archaea. The utility, location of the pipe rig, pipe material and stagnation all had a significant influence on the plumbing microbiome composition, but the utility source water and treatment practices were dominant factors. Examination of 21 water chemistry parameters suggested that the total chlorine concentration, pH, P, SO_4_^2-^ and Mg were associated with the most of the variation in bulk water microbiome composition. Disinfectant type exerted a notably low-magnitude impact on microbiome composition. At two utilities using the same source water, slight differences in treatment approaches were associated with differences in rare taxa in samples. For genera containing opportunistic pathogens, Utility C samples (highest pH of 9–10) had the highest frequency of detection for *Legionella* spp. and lowest relative abundance of *Mycobacterium* spp. Data were examined across utilities to identify a true universal core, special core, and peripheral organisms to deepen insight into the physical and chemical factors that shape the building plumbing microbiome.

## Introduction

Drinking water systems are far from sterile environments, and recent application of molecular methods has revealed surprising diversity in composition [1,2] and function [3]. The microbial ecology of drinking water systems is now understood to play a critical role in a wide range of economic, water management, and health problems, including microbial-induced corrosion [4,5], nitrification in chloraminated systems [6,7], and waterborne disease [8,9].

The portion of the drinking water distribution system within homes and other buildings (i.e., building plumbing or premise plumbing) creates a unique niche for microbial proliferation [10,11]. Relative to the main water distribution system, the surface area to volume ratio is high [12], the water is warm and stagnant during much of the day [13], and disinfectant can decay more rapidly [14], contributing to more prevalent regrowth of microorganisms relative to water mains. These distinctions are important because building plumbing represents the final gateway for exposure of consumers to the microbes inhabiting their drinking water, such as opportunistic pathogens (OPs) (e.g., *Legionella pneumophila*, *Mycobacterium avium*, *Pseudomonas aeruginosa*) [15]. Nonetheless, drinking water regulations are generally focused on water leaving the treatment plant and in the main distribution system and not at the point-of-use where exposure actually occurs.

OPs are now recognized as the primary source of waterborne disease outbreak in developed countries [16,17,18]. In contrast to traditional fecal pathogens, OPs are native to freshwater and drinking water systems [19] and can proliferate in the building plumbing environment [20–24]. Also, many OPs are relatively tolerant of disinfectants [25,26], and broader strategies such as managing the microbial ecology, i.e. a “probiotic” approach, will ultimately be needed to effectively control OPs, as opposed to reliance on disinfectants alone [27]. Thus, a firm understanding of the microbial ecology of building plumbing systems is needed, and recent advances in the application of next generation sequencing technology [28] provides the opportunity to advance towards this goal. Next generation sequencing has successfully captured driving influence of on-site monochloramine to bacterial ecology in a hospital’s hot water system [29,30] and also served to map point-of-use tap water microbiome across seventeen cities along the Arkansas and lower Mississippi rivers [2].

While these pioneering studies provide a glimpse of the vast diversity of microbial communities inhabiting drinking water systems, there are many practical challenges to build a mechanistic understanding of the factors that shape the building plumbing microbiome. First of all, buildings are highly complex, with each essentially representing a unique array of pipe materials [14], configurations [31], and flow-patterns [13]. A question of particular importance is to what degree the building plumbing microbiome is shaped by the influent water quality versus the building plumbing environment itself. Or, to what extent building owners have control over the building plumbing microbiome relative to water utilities. These are very difficult questions to answer based on field studies, as the inherent complexity of building plumbing precludes isolation of factors hypothesized to govern microbial community composition.

This study addresses the above challenges through installation of standardized building plumbing rigs at five water utilities, located in the eastern U.S., and characterization via Illumina sequencing of 16S rRNA gene amplicons. In order to capture the effects of changes in water quality during passage through water mains, one rig was located at or near the drinking water treatment plant and a second rig was installed in a distal portion of the distribution system. The rigs themselves facilitated comparison of pipe material (copper with lead solder, CPVC with brass fitting, copper/lead combined pipe), with uniform flow cycles (ten minutes flow every eight hours) to simulate typical stagnation in household daily water usage (i.e. sleeping hours at night, working hours during daytime). The composition of the building plumbing microbiome was compared across rigs to gain insight into the relative influence of the source water chemistry and treatments, pipe material, water age, and stagnation events.

## Materials and Methods

### Building plumbing rig design and sample collection

Two standardized rigs were installed at each of five potable water utilities (A, B, C, D and E) in the eastern portion of the U.S. At each utility, one rig directly received water from the drinking water treatment plant (WTP) and the other was located in the distribution system (DS) at water ages of ≈ 4.5, 6.5, 0.75, 0.6 and 3 days, respectively [32]. Each rig was constructed with three pipe materials in triplicate: copper with lead solder (referred to as “Copper”), plastic with brass fitting (referred to as “CPVC”), copper/ lead combined pipe (referred to as “Copper/lead”). All rigs were operated under identical flow conditions, mimicking typical household water usage patterns (i.e., 10 L/min for 10 minutes, followed by 7 hours 50 minutes stagnation). The rigs had been acclimated for about 1 year at the time of sampling.

Utility personnel assisted in sampling the rig water between Nov.27^th^ 2012 and Jan.16^th^ 2013. A detailed sampling protocol and pre-assembled sampling kit was provided to each utility to ensure uniformity of sampling. Sample bottles were autoclaved 1000 mL polypropylene wide mouth square bottles (Nalgene^TM^, U.S. Plastic Corp., Lima, OH) containing 26.7μL of 0.5g/mL sodium thiosulfate and 1.4 mL 0.5M EDTA (pH = 8.0) added as preservatives (both sterilized using 0.22 μm filter) to limit damage of microbes and their DNA by any disinfectants or metals in the water.

For each sampling event (3 pipe materials in triplicate and 1 influent for each rig location, a total of 10 samples/rig-event), about 700 mL first flush bulk water samples were collected on three consecutive mornings or flow cycles (Batches 1, 2, 3). Sampling was conducted just prior to scheduled flushing for maximal impact of stagnation. One field blank was included for each sampling batch by transferring 700 mL autoclaved nanopure water, included with the kit, into a sterile sampling bottle after taking 10 samples. Once collected, samples were shipped on ice overnight to Virginia Tech, where they were immediately stored at 4°C and processed within 24 hours upon receipt.

### Site location and water chemistry characterization

All five utilities received surface water as source water. Distinguishing features about the utilities, such as water treatment approach and geographic information, are reported in S1 and S10 Tables. In particular, Utilities A-D used chlorine to provide secondary residual in the distribution system, while Utility E used chloramines. Utilities A and B shared the same source water and both applied conventional treatment processes, with the distribution system at Utility A more than double in size than that of Utility B [32], which provided the opportunity to examine the effect of post-source water factors (see Discussion). Six to seven basic water chemistry parameters were measured throughout the study and on site at the time of Batch 1 sample collection, including: temperature, pH, free chlorine, total chlorine, turbidity, ammonia (chloraminated system only), and corrosion current (copper/lead combined pipes). Prior to Batch 1 sampling, background water chemistry data, including total organic carbon (TOC), SO_4_^2-^, NO_3_^-^, concentration of metal ions were measured at Virginia Tech over a 3 month period [32].

### Sample processing and DNA extraction

Water samples were filtered through 0.22 μm-pore-size sterile mixed cellulose ester filters (GSWP047S0, EMD Millipore, Billerica, MA). The filters were fragmented using flame-sterilized tweezers and placed in 2 mL Lysing Matrix A tubes (MP Biomedicals, Solon, OH). A filter blank was taken at the end of each filtration round by loading it onto the manifold and turning the vacuum pump on for the average time required for samples. Tubes were stored at -80°C, and later subject to DNA extraction using the FastDNA^®^ SPIN Kit (MP Biomedicals) according to manufacturer instruction. A tube blank, which received only reagents and no sample, was included for each DNA extraction round.

### Illumina sequencing

All samples were subjected to PCR amplification [33] using universal bacterial/archaeal primer set 515f/806r, which targets V4 region of 16S rRNA gene [34]. Sample preparation followed the Earth Microbiome Project 16S rRNA Amplification Protocol [35]. Minor changes included using Molecular Biology Grade Water (Quality Biological, Gaithersburg, MD), and QIAquick PCR Purification Kit (QIAGEN, Valencia, CA). Pooled samples were submitted to the Virginia Bioinformatics Institute for paired-end 250 cycle Illumina sequencing (VBI GRL MiSeq sequencing service, Blacksburg, VA). Eight out of 300 samples were precluded due to low yield in PCR products. Field blank, filter blank and tube blank samples were pooled on a “maximum volume” basis instead of equal molar criteria (maximum volume of other samples in the same lane). During the preparation process, all samples experienced identical freeze-thaw cycles.

### Data Analysis

All original DNA sequences and metadata have been deposited to QIITA, under Study ID 10251. PANDAseq was used for joining paired-end sequence reads [36] in Oracle VM Virtual Box version 4.2.12. Stitched reads were pre-filtered based on length (252-255bp) before processed with the Quantitative Insights Into Microbial Ecology (QIIME) version 1.8.0 [33], following an open source online protocol [37]. The *pick_de_novo_otus*.*py* script was used to conduct: 1) *de novo* operational taxonomic unit (OTU) picking, using method uclust_ref [38] with cutoff value of 3%, 2) taxonomy assignment of generated OTUs against Greengenes 13_5 reference database [39], 3) phylogenetic tree construction by FastTree 2.1.3 [40], and 4) OTU table construction. Singletons (defined as an OTU represented by 1 sequence, and appears only once in the whole OTU table) were removed from the OTU table prior to downstream analysis. A total of 35.7 million sequences were retrieved from 292 water samples, with a minimum of 29,238 sequences per sample. Rarefaction to 29,238 sequences was applied to all samples before downstream analysis to minimize impact of uneven sequencing depth.

The Shapiro-Wilk normality test was applied for each water chemistry parameter. Kruskal-Wallis analysis was chosen to assess variance across different utilities. Both tests were performed in R version 3.0.2 [41]. Principal Component Analysis (PCA) was applied for comparing water chemistry parameters across different samples (Primer 6, version 6.1.13). Jackknifed beta diversity based on both unweighted and weighted UniFrac distance matrices [42] were calculated in QIIME to compare microbial composition of different samples. The unweighted UniFrac distance matrix considers presence/absence of each operational taxonomic unit (OTU), while the weighted version includes relative abundance information. Emperor [43] was used to visualize jackknifed beta diversity distance matrices. Analysis of similarity (ANOSIM) and similarity percentage (SIMPER) (both in Primer 6, version 6.1.13) were used to compare similarity/dissimilarity of sample microbiome from the same utility/ utility pair. Indicator species were determined for each utility at species level ({indicspecies}, R). Adonis (permutational multivariate analysis of variance using distance matrices, {vegan}, R) was applied to explore potential impact of single factor or water chemistry parameter. BEST (Bio-Env+Stepwise) analysis (Primer 6, version 6.1.13) was applied to identify a “best” possible combination of water chemistry parameters that explained the largest portion of variation in microbial community composition across samples [44]. This “BEST” set was further chosen for Canonical correspondence analysis (CCA, {vegan} package, R) with taxonomy table at species level. Statistical significance was set at p<0.05.

## Results

### Factors Influencing Water Chemistry

**Fig 1:**
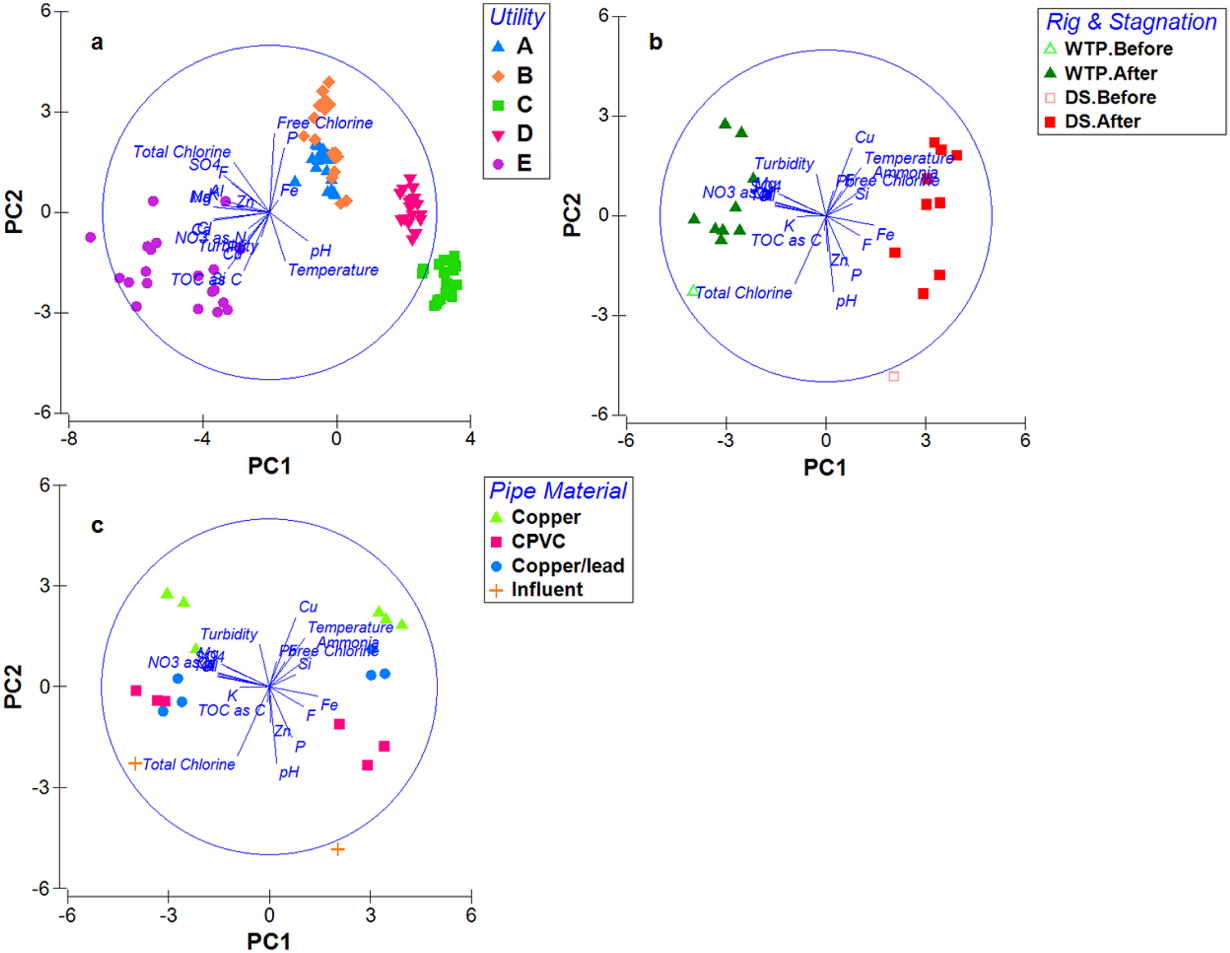
Dissimilarity in water chemistry of samples from different utilities, rig locations, and pipe materials. PCA plot of water chemistry data. Each point represents a water sample, with proximity of points in 2-D space indicative of relative similarities. Principle component (PC) 1 and PC2 are combinations of water chemistry variables that best explained variation among samples. a. samples from five utilities (n = 100 samples), color and shape coded based on utility location; PC1 and PC2 explained 44.2% and 27.3% variation, respectively. b. samples from Utility E (n = 20), shape coded by rig location and color coded by stagnation stage; PC1 and PC2 explained 46.5% and 16.3% each. c. samples from Utility E, color and shape coded by pipe material; PC1 and PC2 explained 46.5% and 16.3% each.

PCA provided a comprehensive comparison of water chemistry data across the utilities (Fig 1) and revealed general trends, indicating that: 1) local water chemistry was distinct at each utility; 2) water chemistry of DS rig samples was distinct from that of WTP rig samples; 3) water chemistry changed during stagnation; and 4) water chemistry changed with pipe materials. The “utility” is an aggregate factor that includes properties of source water, water treatment and distribution process.

ANOSIM results indicated that utility, rig location, and pipe material all contributed significantly to the overall variance in water chemistry data across samples (alpha = 0.05, S3 Table). Among these three factors, utility resulted in the highest global R statistic of 0.713, compared to 0.332 for location of rig at WTP or in DS (nested under utility), and 0.448 for pipe material (nested under utility.rig). Comparing each influent to corresponding effluents, 8-hour stagnation did not have a measureable impact on water chemistry, regardless of pipe material (P = 0.18, nested under utility. rig). The impact of stagnation was noted to be non-uniform for each pipe material (i.e., concentrations of Pb, Cu, Zn), which could have masked the overall impact of stagnation on water chemistry.

All water chemistry parameters varied across utilities (P < 0.05, S4 Table), except for Zn. Total chlorine concentration was highest at Utility B (1.16±0.38 mg/L) and lowest at Utility C (0.23±0.28 mg/L). In most cases, disinfectant decay was apparent in the influents to the DS rigs relative to the WTP rigs based on examination of data 3-month prior to sampling (S1 Fig). An exception was the higher total chlorine levels observed in the influent to the DS rig relative to WTP rig at Utility B (~0.5 mg/L), which is likely the result of fluctuation in water treatment and chlorine dosing, along with the fact that there was a 6.5 day water age delay between the DS and WTP rigs. The highest pH occurred at Utility C (9–10), with other utilities in the more typical, EPA recommended range of 6.5–8.5 (S2 Fig). Certain parameters, including concentrations of Pb, Cu, and Zn after stagnation, were mostly influenced by pipe material, rather than utility (S4 Table). For instance, Pb concentration was usually highest in copper/lead combined pipe (992.6±1773.9 ppb, with 190.0±498.9 ppb in copper pipe and 3.1±3.7 ppb in CPVC pipe); Cu concentration was highest in copper pipe (252.6±268.5 ppb, with 139.2±163.6 ppb in copper/lead combined pipe and 33.8±34.7 ppb in CPVC pipe); and Zn concentration was highest in CPVC pipe (141.3±126.8 ppb, with 32.9±79.0 ppb for copper pipe and 19.2±32.1 ppb for copper/lead combined pipe). “Spikes” of turbidity mostly (9 out of 10 values larger than 1 NTU) occurred in copper pipe and copper/lead combined pipe.

Utility A and B, which shared the same source water, had the most similar water chemistry relative to the other utilities (Fig 1), with a few distinctions noted. For instance, Utility B had higher total chlorine concentration (1.16±0.38 mg/L), but lower pH (7.3±0.1) compared to Utility A (total chlorine 0.73±0.24 mg/L, pH 8.6±0.1). Also, Utility B had about twice the phosphorus concentration as Utility A (250.9±21.2 ppb vs 135.1±5.7 ppb).

### Microbiome Composition

#### Microbial community composition

**Fig 2:**
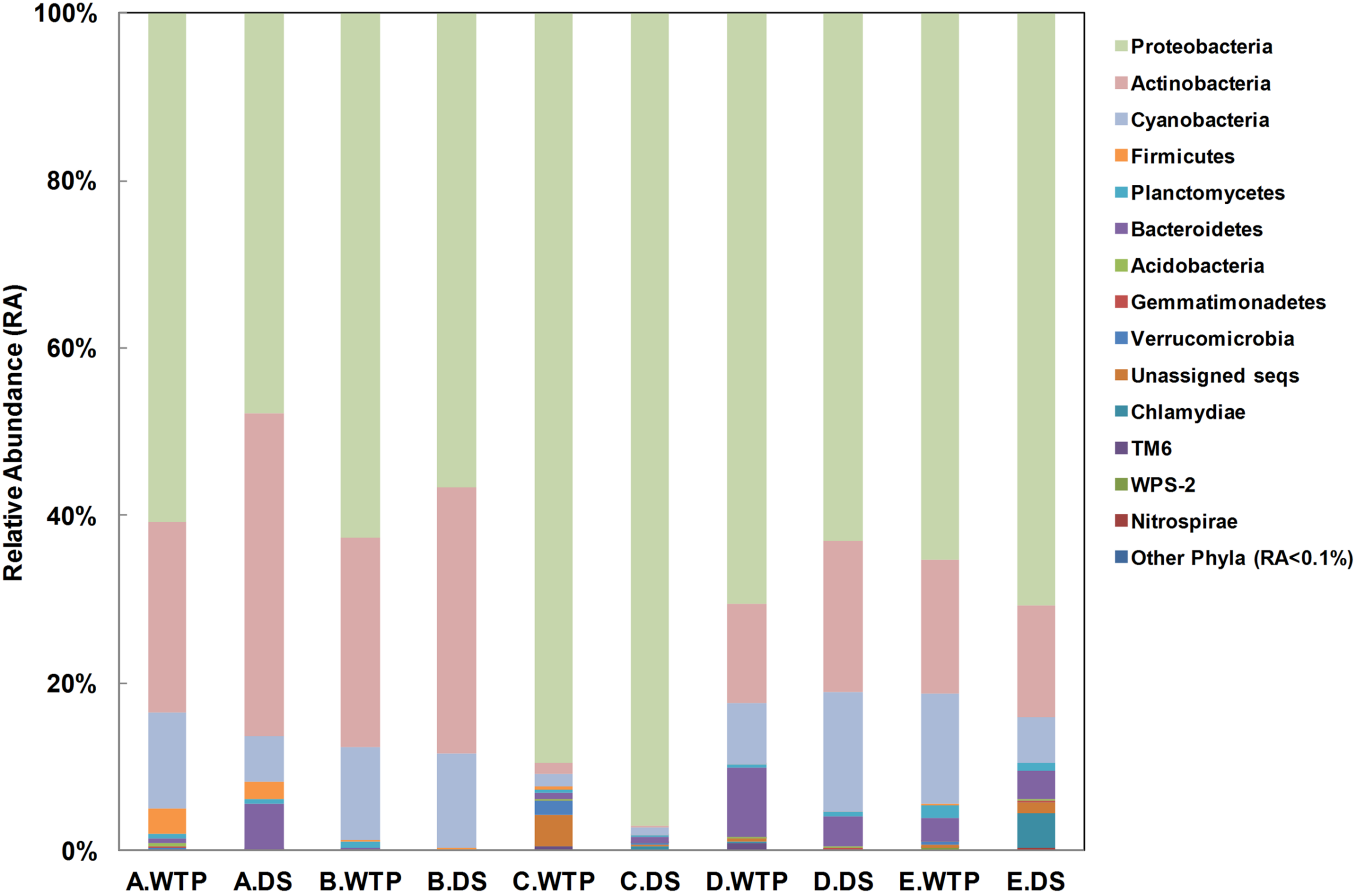
Microbiome taxonomy composition of samples from each rig (phylum level). Data were combined across all 27 pipe samples for each rig. Relative abundance was calculated as the ratio of sequences. Phyla with relative abundance less than 0.1% were combined into “Other Phyla (RA<0.1%)”.

A total of 3 archaeal and 37 bacterial phyla were detected across all samples, with 0.002% archaeal sequences, 99.3% bacterial sequences and 0.7% unclassified sequences. Four dominant (i.e., >1% relative abundance) bacterial phyla accounted for 96.9% of the total sequences: *Actinobacteria* (17.6%), *Bacteroidetes* (2.6%), *Cyanobacteria* (8.2%) and *Proteobacteria* (68.6%) (Fig 2).

#### Genera Containing Opportunistic Pathogens (OPs)

Genera containing OPs were widely detected in samples across the five utilities (Table 1). *Legionella* spp. were detected in samples from all five utilities, except from Utility D, with the highest frequency of detection in Utility C samples. At Utility C, *Legionella* spp. were detected in 29 out of 30 WTP rig samples and 18 out of 29 DS rig samples, whereas all detections in Utility E occurred in DS rig samples.

**Table 1:** Frequency of detection of genera containing OPs across the five utilities (n = 60, 54, 59, 60, and 59 samples for Utility A, B, C, D and E, respectively).

	A	B	C	D	E
***Legionella* spp.**	1.7%	7.4%	78.0%	0.0%	13.6%
***Mycobacterium* spp.**	100%	100%	98.3%	100%	100%
***Pseudomonas* spp.**	96.7%	48.1%	86.4%	70.0%	93.2%

*Mycobacterium* spp. were detected in all samples except one. The relative abundance of *Mycobacterium* spp. was noted to be 2 orders of magnitude lower at Utility C (7.4×10^−4^) relative to the other four utilities (>0.14). The frequency of detection of *Pseudomonas* spp. was > 70% of samples across all utilities, with the exception of Utility B. Utility B samples had the lowest relative abundance (0.01%) of *Pseudomonas* spp., while highest relative abundance was observed in Utility A samples (1.54%).

#### Comparison of the Microbial Community Composition

**Fig 3:**
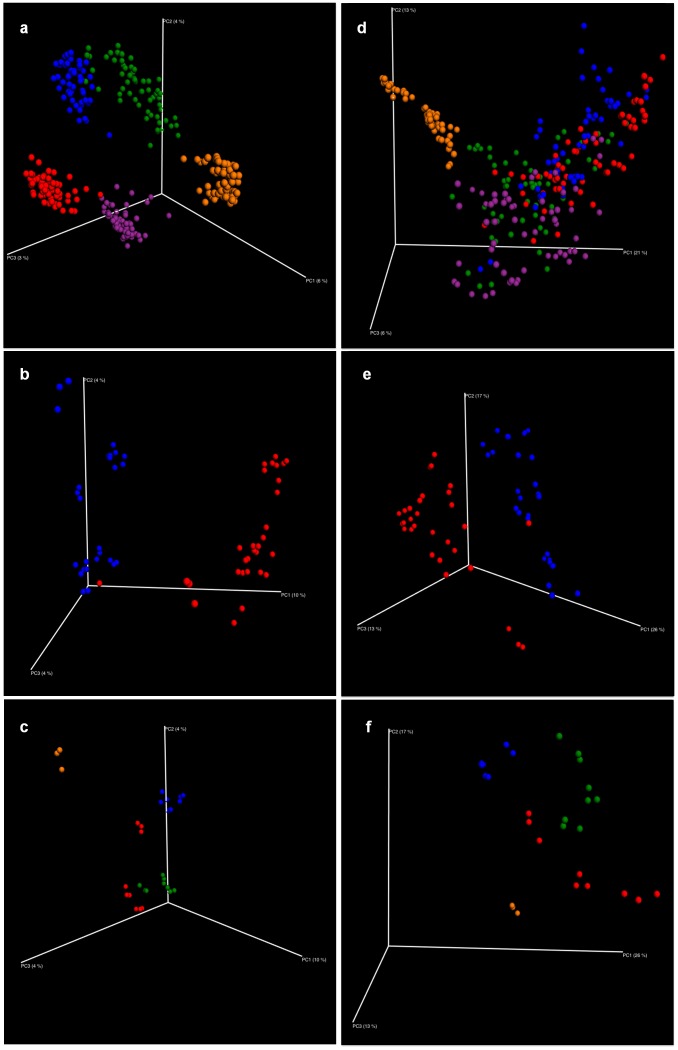
Dissimilarity in microbiome composition of samples from different utilities, rig locations, and pipe materials. 3-D beta diversity plots derived from jackknifed unweighted (a, b, c) and weighted (d, e, f) UniFrac distance matrices, color coded by: 1) utility (a and d), all samples (n = 60, 54, 59, 60, 59, color = red, blue, yellow, green, purple for A, B, C, D, E, respectively); 2) rig location (b and e), Utility E samples (WTP in blue, n = 29; DS in red, n = 30); 3) pipe material and stagnation (c and f), Utility E, WTP rig samples (n = 9, 9, 9, 3, color = blue, red, green, brown for Copper, CPVC, Copper/lead, and influent, respectively).

3-D beta diversity plots constructed from both unweighted and weighted UniFrac distance matrices illustrated several key conclusions about microbial community composition (Fig 3): 1) samples from the same utility were more similar to each other in terms of presence/absence of specific OTUs, relative to other utilities, 2) within the same utility, samples from the WTP and DS rig were distinct, 3) within the same rig, pipe material and stagnation both had an influence. It is of interest to note that the distinction in microbial community composition across the utilities was most apparent based on the unweighted UniFrac matrix (which considers presence/absence of OTUs), not the weighted UniFrac distance matrix (which considers relative abundance in addition to presence/absence of each OTU). However, both distance matrices indicated similar 3-D patterns with respect to effect of rig location and pipe material within each utility/rig.

ANOSIM pair-wise tests (S5 Table) based on weighted and unweighted UniFrac distance matrices confirmed the above 3-D patterns. A global R value greater than 0.75 indicates microbial communities strongly different in composition, whereas an R value between 0.5 to 0.75 indicates some compositional overlap. For unweighted UniFrac distance matrix comparisons, a global R value greater than 0.75 was obtained for all paired utility comparisons, except for B vs D. For weighted UniFrac distance matrix comparisons, paired comparisons between C and the other four had an R>0.75, while all utility pairs among A, B, D, and E had an R<0.5, suggesting large compositional overlap.

Utility, rig location, pipe material and stagnation each had a significant impact on microbiome composition, with utility as the master factor (Table 2). Utility, rig, pipe material and stagnation together explained the greatest dissimilarity in microbiome across samples, followed by utility and rig, then utility only. Within each set of utility samples, relative importance of rig location and pipe material varied (S6 Table). All factors showed larger magnitude of impact when calculated based on weighted UniFrac distance matrix (Table 2). Sample Batch had a statistically significant, but low magnitude impact on microbiome composition, and thus Batch 1 samples were further examined to explore the association between microbiome composition and water chemistry.

**Table 2:** Impact of various factors on the microbiome across all samples (n = 292).

Factor	Strata	Unweighted UniFrac		Weighted UniFrac	
		R^2^	P	R^2^	P
**Utility & Rig & Pipe & Stagnation**		0.339	0.001	0.702	0.001
**Utility.rig**		0.220	0.001	0.520	0.001
**Utility**		0.157	0.001	0.387	0.001
**Rig**	Utility	0.013	0.001	0.034	0.001
**Pipe Material**	Utility.rig	0.014	0.001	0.030	0.001
**Stagnation**	Utility.rig	0.005	0.001	0.015	0.001
**Batch**	Utility.rig.pipe	0.005	0.001	0.003	0.008
**Disinfectant type**		0.032	0.001	0.066	0.001

Adonis analysis was applied using package “vegan” from R, with permutation = 999. Unweighted UniFrac considers presence/absence of each OTU, while weighted UniFrac also considers relative abundance of each OTU. “Strata” were defined based on sampling design as the overarching factor, with the subsequent hierarchy of factors derived according to relative magnitudes of impact on the microbiome. Further permutations were constrained within a given stratum. For example, when Utility.rig (utility and rig location) was set as the stratum for the purpose of examining the impact of stagnation, samples from the same Utility.rig were pooled and randomized, but samples across different Utility.rig combinations were not.

#### Microbial classes driving similarity and dissimilarity

SIMPER (Similarity Percentages- species contribution, Primer 6) analysis was applied to explore microbial classes that drive microbial similarity within the same utility, and dissimilarity across different utilities (S7 Table). In general, *Alphaproteobacteria*, *Actinobacteria*, *4C0d-2*, *Betaproteobacteria*, and *Gammaproteobacteria* were the main contributors in driving microbiome similarity within each utility and dissimilarity across different paired utilities. These were also top 5 abundant classes among all sequences combined.

#### Candidate indicator taxa for each utility

Indicator taxa are genera (or lowest taxonomy levels) whose presence/absence and relative abundance respond to particular environmental variables or specific environments and therefore best characterize a particular group of samples. With respect to utility location, indicator taxa were: 1) mostly found in that particular utility and 2) present in the majority of samples from that utility [45]. For each utility, the top 10 candidate indicator taxa with the highest indicator value and statistical significance <0.05 are reported in S8 Table. For Utility B, all indicator genera fell within the top 52% of genera ranked from highest to lowest relative abundance. For the other four utilities, all but one of the indicator genera were among the top 20% of genera ranked according to relative abundance.

#### “Core” microbiome

**Fig 4:**
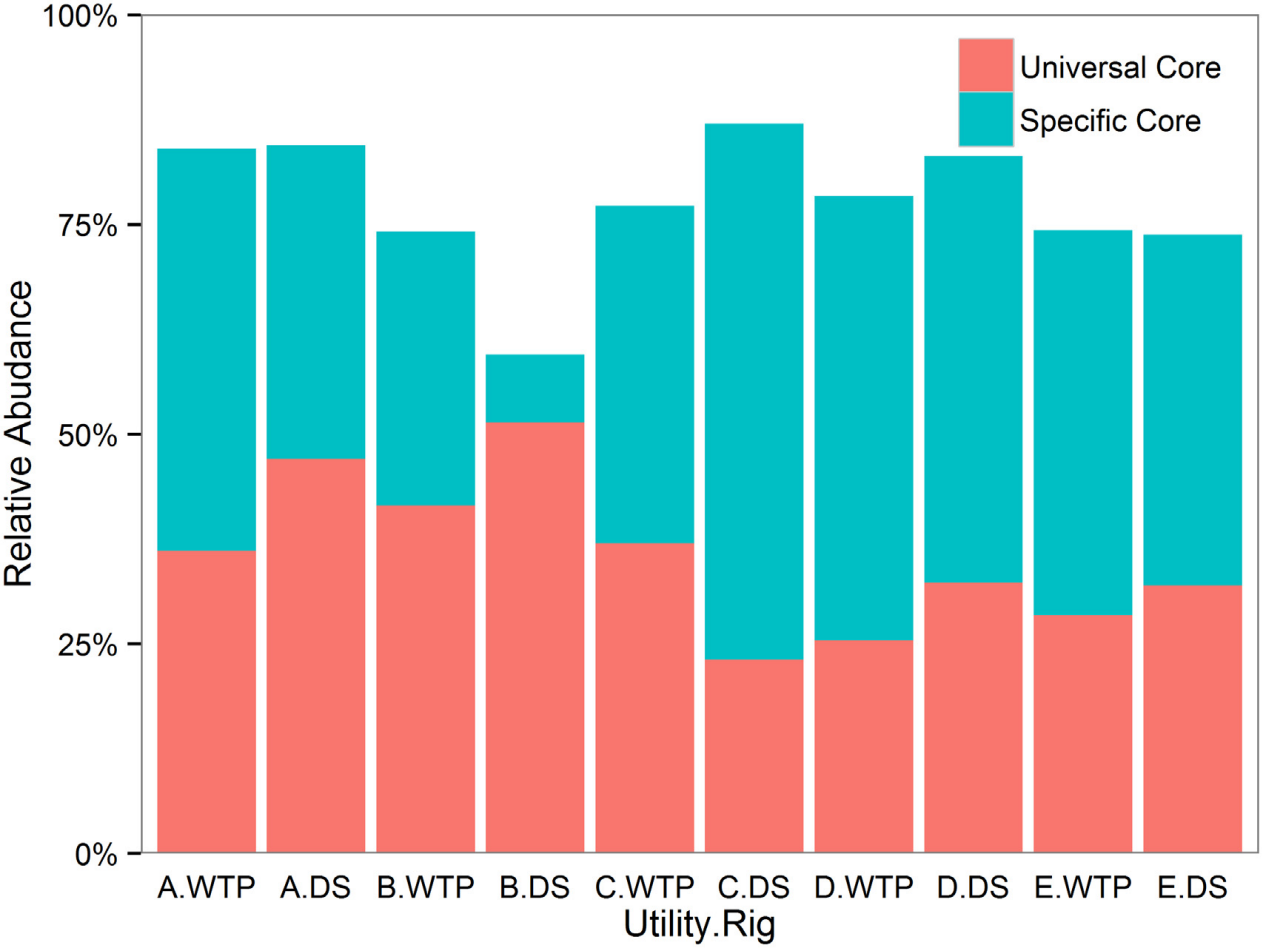
Core OTU comparison across each rig location at each utility. Relative abundance was calculated by normalizing number of core OTU sequences to the total number of sequences within specific Utility.Rig combination. The universal core is defined as OTUs shared among all samples, while the specific core consists of OTUs shared within each Utility.Rig, but not across all samples.

One question of interest is if a “core” drinking water microbiome can be identified [46]. The core microbiome is often defined as OTUs shared above a selected threshold percentage of samples within a certain category (*e*.*g*. samples from same utility, samples collected on same day) [47]. In this study, the threshold percentage was set at 100% and category set defined as utility and rig location together (termed “Utility.Rig”). Core microbiome for each utility & rig combination were further classified into two parts- a universal core (shared among all samples from five utilities combined), and a specific core (OTUs shared among samples within a specific Utility.Rig category, but not in all samples). Size of core microbiome varied, on average 78.0±14.1% of the total sequences (Fig 4).

A second question is whether the “core” microbiome for each utility/rig category are essentially representative of the microbiome of all samples. The Mantel test was applied to compare UniFrac distance matrices constructed from core microbiome by Utility.Rig and the total microbiome. There was a significant and strong positive correlation between corresponding distance matrices (unweighted UniFrac, R statistic = 0.837; weighted UniFrac, R statistic = 0.907; P = 0.001), implying potential of a core microbiome capturing main patterns across all samples.

### Association between microbiome and water chemistry

#### Association between microbiome and individual water chemistry parameters

Most water chemistry parameters were associated with significant differences in microbiome composition (both weighted and unweighted UniFrac distance matrices), although the strength of association was much less than the aggregate association of the microbiome with utility (S9 Table). Among 21 parameters, turbidity did not have a statistically significant association with the microbiome composition, while NO_3_^-^ and Pb only indicated a significant association when using the unweighted UniFrac distance matrix (presence/absence of microbes). The remaining parameters all were more strongly associated with differences in the microbiome composition when applied to the weighted UniFrac distance matrix. Only six parameters had a strength of association larger than 0.1 (weighted UniFrac).

#### Association between microbiome and multiple parameters

The influent and effluent waters of the pipe rigs, including rigs at Utilities A and B, which shared the same original source water, were distinct in both water chemistry and microbiome composition. Of all possible combinations of water chemistry parameters, total chlorine concentration, pH, phosphorus (P), sulfate (SO_4_^2-^) and magnesium (Mg) together explained the greatest dissimilarity of microbiome composition of corresponding samples across the five utilities (weighted UniFrac distance matrix) (Table 3). These parameters also displayed higher individual association with the microbiome (S9 Table). If only considering presence/absence of microbes (unweighted UniFrac distance matrix), pH and Mg played predominant roles (Table 3).

**Table 3:** Association between microbiome and lumped water chemistry variables.

	Unweighted UniFrac	Weighted UniFrac
**“BEST” combination**	pH, Mg	Total chlorine, pH, P, SO_4_^2-^, Mg
**Rho statistic**	0.741	0.501

BEST analysis was conducted using Primer 6. Rho statistic represents “association strength”, ranging from 0 to 1 with rho>0.5 to be generally considered a strong association. Permutation = 99.

**Fig 5:**
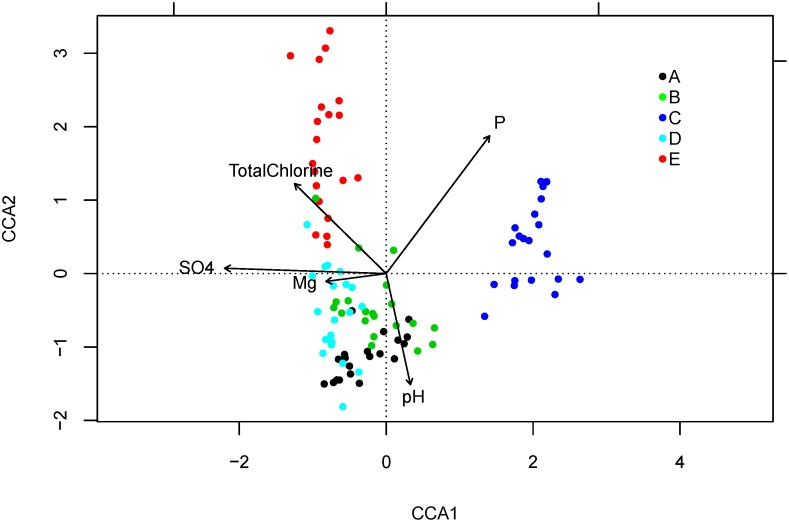
Microbiome composition (genus level) in association with “BEST” water chemistry parameters of Batch 1 samples. Each point represents microbiome of one sample. CCA1 and CCA2 each explained 43.4% and 21.1% of all five constrained axes generated by Canonical Correspondence Analysis (CCA).

Canonical Correspondence Analysis (CCA) was applied to visualize how microbial taxonomy composition (at genus level) was associated with the “BEST” set of water chemistry parameters, including total chlorine, pH, P, SO_4_^2-^, and Mg (Fig 5). The portion of variance in microbiome taxonomy that could be explained by the “BEST” set was 20.2%. The pattern in Fig 5 was in accordance with water chemistry data based on the fact that: 1) Utility E samples were differentiated from others due to highest total chlorine concentration; 2) Utility C samples had the lowest P (near to 0 compared to others at hundreds ppb level), highest pH (up to 10 compared to others in neutral range), lowest Mg (less than 10% compared to others) and lowest SO_4_^2-^ (~20% compared to Utilities A, B and E); and 3) Utility D samples had ~16% of the SO_4_^2-^ concentration compared to Utilities A and B, with samples from these three utilities varying along a gradient of SO_4_^2-^.

## Discussion

### Utility is the overarching factor for both water chemistry and microbiome composition

The unique, standardized and replicated pipe rig design employed in this study enabled a controlled comparison of several variables on the ultimate microbial composition at the tap. Among the four main factors that were the focus of this study: utility, rig location, pipe material and stagnation all had a significant influence on water chemistry and microbial composition. Among these, the utility itself had an overarching influence (Table 2 and S3 Table). Notably, though Utilities A and B shared the same source water, the chemistries and microbiome compositions of the corresponding rigs were distinct, emphasizing the importance of water treatment and distribution in shaping the building plumbing microbiome. This finding is consistent with a recent lab-scale study, in which water generated from simulated distribution systems representing different pipe materials, disinfectants, and water ages was the dominant influence on the composition of the microbial community and occurrence of OPs in simulated household water heaters, rather than the warm, stagnant environment of the heater itself [48].

A recent Netherlands survey on distribution systems corresponding to four different groundwater treatment plants (flushed water sample, Illumina sequencing of 16S rRNA gene amplicons) also indicated that utility, over distance (0.4–35 km to treatment plant) and time (3 sampling events in 17 months), was the predominant factor driving microbial community composition in drinking water [49]. Interestingly, potable water is not subject to secondary chlorination in the Netherlands, indicating that other subtleties of the aquifer and water chemistry, besides disinfectants, can drive significant differences in the corresponding microbial community.

In addition to source water properties and the water treatment/distribution processes, the term “utility” includes local geology, regional watershed characteristics, climate, and weather or other events at the time of sampling. Geographical location is likely to also be important [50,51], in addition to historical events. One study proposed possible historical pig manure application and agricultural runoff to explain the concurrence of high nitrate concentration and significant enrichment in *Erysipelothrix* spp. [2].

### Impact of source water vs water treatment and distribution process

Source water type (surface vs groundwater) and ratios of blends of different source waters was found to influence abundance and proportion of certain taxa, *e*.*g*. sulfur metabolism related microbes were enriched in groundwater sources [2,52]. In this study, all source waters were surface water (either river, lake, or reservoir); however, the water chemistry into and out of the building plumbing rigs still displayed distinct features, implying that the influence of water chemistry goes beyond broad classification of surface water versus groundwater.

Of special interest is whether the drinking water treatment and distribution process could override source-water characteristics in terms of the microbiome that shows up at the tap. Microbial composition has been observed to shift during the various stages of treatment from source water [53]. One other study did indicate that moving downstream of the treatment application, the source water (surface water from River Murray) outweighed impact of different treatment processes (conventional coagulation, magnetic ion exchange resin plus conventional coagulation, magnetic ion exchange resin plus conventional coagulation and granular activated carbon, and membrane filtration) on microbial composition of the biofilm at a distance as short as 1km [54].

Utilities A and B shared the same source water and similar water chemistry (Fig 1) and their microbiome compositions (weighted UniFrac, Fig 3) were correspondingly most similar amongst all utility pairs, indicating an influential role of source water. Nonetheless, there were notable differences in the microbiome composition and water chemistry features between Utilities A and B, which likely related to differences in water treatment and distribution. Specifically: a) Utility A mostly used alum as coagulant with occasional application of ferric sulfate, while Utility B used ferric sulfate exclusively; b) for corrosion control, Utility A employed lime softening and caustic, while Utility B used blended phosphate and caustic; c) Utility A applied granular activated contactors along with traditional treatment, and UV disinfection as an extra safe-guard. The above differences most likely induced variation in several key water chemistry parameters (e.g. pH, total chlorine, phosphorus) for treated waters from Utilities A and B. For instance, elevated phosphorus level in Utility B water samples most likely arose from application of phosphate for corrosion control. The influenced parameters were all among the “BEST” set that identified parameters most strongly associated with differences in microbiome composition across utilities. While the microbiome compositions of Utilities A and B were similar by weighted UniFrac method (with relative abundance), they were distinct by unweighted UniFrac method (presence/absence only). This discrepancy indicates that the above differences in water treatment and distribution between the two utilities were especially influential on rare taxa, the importance of which should not be overlooked (e.g., OPs are typically “rare”). Thus, while source water is likely an overarching factor, water treatment and distribution management choices available to utilities alter the key water chemistry components and ultimate microbiome composition in the building plumbing.

### Influence of location in the distribution system, stagnation, pipe material

Comparison of the WTP and DS rig samples was of particular interest, as this provides insight into the role of water age in shaping the building plumbing microbiome. In this study the water age entering the DS rigs ranged from 0.5–7 days. In a laboratory simulation of a water distribution system, water age (ranging from 1 to 10.2 days) was observed to significantly shape both the microbiome and key water chemistry parameters (disinfectant residual, total organic carbon and dissolved oxygen) [55,56]. This study confirmed that water quality at the tap varied based on location in the distribution system, even with as little of difference of 0.5 d water age, and provided insight into the relative degrees of associated microbial shifts.

To the knowledge of the authors’, this is the first controlled study of the impact of a typical building plumbing stagnation period, ~8 hr, on the microbial composition of the tap water. In terms of water chemistry, stagnation dramatically influenced free chlorine, and selectively influenced Pb, Cu and Zn concentrations for certain pipe materials. Water samples after stagnation yielded 6–13 more phyla compared to corresponding influents (before/after stagnation: A 10/22, B 9/16, C 13/26, D 14/20, E 16/29). The increase suggested potential “seeding” from building plumbing biofilm, or regrowth of rare species above the detection limit, likely as a result of disinfectant decay and the magnified influence of biofilms in the small diameter pipe. Overnight stagnation was reported to result in cell regrowth and a shift in 50–100% of the microbial community composition in drinking water samples (bulk water) from different households in a non-chlorinated water system [57]. In this study, a significant effect of stagnation was observed in all utilities, except for Utility B. It was noted that samples after stagnation were still more closely related to the corresponding influent to the rig than to the stagnated water from pipes of the same type from other rigs. Similar spatial stability in drinking water treatment plant and distribution systems have been reported in other studies [49,58–60].

Pipe material exhibited a significant effect on 4 out of 21 water chemistry parameters: Pb, Cu, Zn and turbidity (S4 Table). These parameters are highly pipe material-specific: as Pb was released mostly in copper/lead combined pipe, less in copper pipe (i.e. lead solder) and only slightly in CPVC pipe; Cu was released mostly in copper/lead combined pipe and copper pipe; and Zn was released mainly from brass fitting on CPVC pipe. Turbidity likely was a byproduct associated with metal particles and biofilm released from metal pipes. Pipe material appeared to be strongest determinant of the microbiome composition at Utility E, outweighing the impact of the rig location at the WTP or in the DS (S6 Table). Pipe materials are known to influence biofilms in terms of biofilm density [61], bacterial diversity [62], formation potential [63] and formation rate [64], which could indirectly influence bulk water microbiome composition.

### What are the predominant water chemistry parameters?

Among the “BEST” water chemistry parameter set across multiple utilities, total chlorine concentration [59], pH [60], P and SO_4_^2-^ together [52] have all been previously reported as strongest determinant(s) of the microbial composition of drinking water. A few studies examine the importance of Mg to the drinking water microbiome, and we speculate that Mg in this study is an indicator of salinity (S3 Fig), which is widely reported to be the strongest determinant of microbial community structure in aquatic systems [65–67]. CCA analysis further suggested the potential for complex interrelationships among water chemistry parameters (Fig 5), indicating “cooperative,” rather than individual, influence on the microbiome. For example, disinfectant efficacy varies among target microbes, while pH governs the relative proportions of hypochlorous acid versus hypochlorite, which also have differing efficacies.

Surprisingly, TOC was not among the “BEST” variables identified as having influence on the microbial composition. The assimilable portion of the TOC [68,69] and phosphorus [69,70] have been noted as important limiting nutrients for microbial growth in drinking water distribution systems. In a recent study of simulated household water heaters, total heterotrophic plate count bacteria correlated with TOC, but ranged within the same order of magnitude (2,000 μg/L to 15,000 μg/L) [71]. In the present study, TOC was generally low (1035±603 μg/L) across all utilities and thus likely was not in an ideal range to capture variability.

In contrast to previous reports of the dominant effect of chloramine versus chlorine in shaping microbial composition [3,29,59,72], we observed limited effect of disinfectant type on microbiome composition of the building plumbing microbiome relative to the other factors investigated (Table 2). Greater dissimilarity in microbiome composition occurred among chlorinated systems, relative to paired comparisons with the chloraminated utility (S5 Table). Disinfectant type appeared to impose stronger influence on rare taxa [2], an effect that would have limited resolution with the weighted UniFrac distance matrix approach (relative abundance included in calculation). Again, while disinfectant type clearly has an effect, this study indicates that the utility as a whole is the overarching factor. We further speculate that the precise predominant water chemistry parameters vary within each utility, and are not necessarily the same across utilities. It is also noteworthy that the most influential water chemistry variables might change through seasons or due to operational changes [52].

### Several abundant taxa comprised the majority of microbiome

A total of 22, 16, 26, 20, and 29 different phyla were recovered for Utility A, B, C, D, and E, respectively, while previous drinking water studies have recovered: 9 to 16 [60], 26 [2], 26 [72], 11 [73], and 15 [74]. Consistent with prior drinking water surveys, the majority of total sequences were assigned to a few dominant phyla [2,52]. The dominant phyla observed in this study, *Actinobacteria*, *Bacteroidetes*, *Cyanobacteria* and *Proteobacteria*, have been commonly reported in other drinking water distribution systems [2,58,75]. Such high similarity in dominant phyla across multiple study sites could be attributed to unique aspects of the drinking water environment, including similar micro-environments (i.e. standardized rig setting, water treatment plant) [2]. Within the Proteobacteria phylum, other studies have also noted the overall predominance of the *Alphaproteobacteria* class in water mains [3,76,77], and a shift within proteobacteria to *Alphaproteobacteria* during winter season [52].

### Genera containing opportunistic pathogens (OPs)

Amplicon sequences obtained from Illumina sequencing are short (~250 bp, without primers or barcodes), thus they have limited taxonomic resolution and cannot definitively identify pathogens. However, several genera known to contain OPs were identified, and examining these patterns could provide insight into the behavior of pathogenic members. Water chemistry, microbiome composition, and occurrence of genera containing OPs appeared to have a complex interrelationship [19,27]. *Legionella* is acid-tolerant [78] and most isolates have been obtained from environmental sources in the pH range of 2.7–8.3 [79]. Further, it has been reported in prior studies that *Legionella pneumophila* has reduced viability and cultivability at higher pH [80,81]. Thus, hypothetically, the high pH level at Utility C (9–10) would have negatively influenced the occurrence of *Legionella* spp. In contrast, Utility C had the highest frequency of detection of *Legionella* spp. One possible explanation is that the relatively low disinfectant residual levels, which have been associated with high frequency of detection of *Legionella* spp. [82,83], offset the potential inhibitory effects of elevated pH. *Legionella* spp. had higher frequency of detection in WTP rig samples (28 out of 29) relative to DS rig samples (18 out of 30), while disinfectant residual level in the DS rig was ~0.5 mg/L lower than that of WTP rig samples. This suggested there are not necessarily simple linear relationships between *Legionella* spp. and isolated water chemistry parameters in real-world pipes. It is interesting that Utility C samples also had the lowest relative abundance of *Mycobacterium* spp., indicating the potential for competition between the two OPs-containing genera, or environmental selection favoring *Legionella* spp. over *Mycobacterium* spp., as higher pH is expected to result in more effective inactivation of the latter [25].

### Nitrogen-cycling bacteria

Ammonia-oxidizing bacteria (AOB) and nitrite-oxidizing bacteria are a nuisance in chloraminated drinking water distribution systems because they catalyze decay of chloramine disinfectant [6,7,84]. In this study, nitrite-oxidizing bacteria were primarily *Nitrospira* spp., which was also an indicator genera for the chloraminated utility (Utility E). Predominance of *Nitrospira* spp. over *Nitrobacter* spp. (latter in 5 out of 59 samples) might be explained by advantages of k-strategists over r-strategists in oligotrophic water: the former exhibits a low maximum specific growth rate, but is adapted to low nitrite and oxygen concentrations [85]. Common AOB genera, including *Nitrosomonas*, *Nitrosoccocus* and *Nitrosospira* [86], were not detected. However, the potential AOB-related family, *Nitrosomonadaceae*, was detected at a relative abundance of (1.3±0.8%) across all Utility E samples, which was the highest frequency of detection among the five utilities. *Candidatus Brocadia*, mainly detected in freshwater/estuary [87], was detected in 1 sample only, suggesting the possibility of autotrophic bacteria using nitrite to oxidize ammonia under anoxic conditions [88,89].

### Candidate indicator taxa

Indicator genera suggested potentially important metabolic processes occurring in the rigs. *Hydrogenophaga*, an indicator genus at Utility B, is a hydrogen-oxidizer [90]. At Utility C, *Limnobactor* is associated with sulfur-oxidation, with certain species able to oxidize thiosulfate [91]. *Methylocaldum* spp. are mesophilic to thermophilic obligate methanotrophs, using methane and methanol as carbon and energy source [92]. *Hyphomicrobium* spp. are methylotrophic microbes widely detected in various habitats including groundwater and fresh water [93].

Indicator taxa could also reflect source water background. As an indicator for Utility D, *Polaromonas* genus is psychrophilic and predominant in glacial, periglacial and high-elevation environments [94], which is concordant with the source water feature and geographic location of Utility D. Also, indicator taxa provide preliminary insight into potential risk. In Utility C, *Legionella* spp., which contains *Legionella pneumophila*, was one of the indicator genera. At Utility A, *Staphylococcus* was the top ranked indicator genus. While *Staphylococcus* has high occurrence on human skin [95], its absence from any of three field blanks implies little likelihood of originating from contamination by sampling personnel. A prior survey noted that 6% of individual rural western drinking water wells cultured positive for natural sources of *Staphylococcus aureus* [96].

### Quality control indicators for sampling low biomass water

To maximize yield of DNA and consider repeatability of sampling procedures, each rig was sampled three times within a three-day period. Sampling batch exerted little to no influence when the microbiome composition was compared within each utility (S6 Table) and across utilities (Table 2). Long-term temporal stability is also sometime observed, as was the case in a 17-month study of an unchlorinated distribution systems in the Netherlands [49]. However, a study in Michigan indicated dramatic seasonal patterns [52].

Of all 39 blank samples (30 field blanks, 4 filter blanks and 5 tube blanks), only 3 field blank samples (all from WTP rig at Utility A) yielded detectable sequences: 943, 1162, and 1212 sequences (S2 Table). The proportion of blank sequences to the minimum number of sequences obtained among samples was less than 7%. Thus, extent of potential “contamination” sequences was considered to be small in this study.

### Core microbiome insight into source of the building plumbing microbiome

A question of interest is whether a healthy drinking water environment would “select” for a stable core microbiome. This study encompasses 10 different geographical locations (Utility.Rig), with the “universal core” defined as OTUs shared in all samples across the five utilities. The universal core contained five OTUs, three of which were also detected in all three field blanks that yielded sequences, but at very low abundance as mentioned above. A “special core” was defined as OTUs shared within samples from the same Utility.Rig combination, but not represented in the universal core. OTUs not belonging to either core are termed here as “peripheral”. Size of the universal core and special core within each Utility.Rig category varied, together constituting over 55% of the total microbiome. Moreover, Mantel test results indicated that the core microbiome was representative of the total microbiome.

Existence of a true universal core, special core, and peripheral organisms implies potential differences in dispersal limitations. For instance, the universal core might be linked with high dispersal and colonization rate, high tolerance to stress, and the ability to compete with indigenous microbes [51]. Dispersal limitations were recently suggested for the planktonic portion of the water distribution system microbiome [52]. The composition of the drinking water microbiome can vary across different phases (e.g., bulk water, biofilm, loose deposits and suspended solids) [74], stages (e.g., source water, treatment plant, distribution system, built environment/building plumbing) [46], and seasons [52]. Such high complexity would inevitably lead to a core microbiome that shifts as a function of time and location [52]. The core microbiome concept could be useful in further studies in elucidating the driving factors that dictate the composition of the microbiome at the tap [46].

## Conclusion

This study employs a unique standardized building plumbing rig design to provide controlled, replicated comparison of potential factors contributing to the microbial composition in drinking water at the tap, questions that are confounded by the complexities faced in field studies. Overall, it was observed that the utility itself was the overarching factor in shaping the building plumbing microbiome, while location of the rig in the distribution system, plumbing material, and 8-hr stagnation events also had a significant influence. Total chlorine concentration, pH, P, SO_4_^2-^ and Mg together explained greatest variation in microbiome at multi-utility level, however within each utility such “BEST” set might change. This study suggests that factors under control of the utility, including physical/chemical properties of the water and prior treatments, drive the composition of the building plumbing microbiome, including the occurrence of opportunistic pathogens. However, factors under control of building owners also clearly have an influence. These findings have important implications for water engineering and management, helping lay the groundwork needed to identify critical factors that may be manipulated in the future to beneficially manage the building plumbing microbiome.

## Supporting Information

S1 Fig Total chlorine concentrations of samples from water treatment plant (WTP) and distribution system (DS) rigs. Each rig contains 9 pipe samples (triplicates of the three materials) following ~8hrs stagnation. Squares with cross inside are influent water samples, short lines represent average influent total chlorine concentration during 3-month prior to sampling event. Utilities A-D delivered chlorinated water, while Utility E delivered chloraminated water. The total chlorine concentration range was from 0.04–1.88 mg/L.
(TIFF)

S2 Fig pH of samples from water treatment plant (WTP) and distribution system (DS) rigs. Each box contains 9 pipe samples from same rig (triplicates of the three materials) following ~8hrs stagnation. Squares with cross inside are influent water samples, while short lines are average influent pH values during 3-month prior to sampling.
(TIFF)

S3 Fig Positive correlation between total dissolved solids and Mg concentrations. Total dissolved solids were calculated using water chemistry data, representing salinity of drinking water.
(TIF)

S1 Table Geographical distances among five utilities. (DOCX)

S2 Table Taxonomy composition of 3 field blanks from Utility A, WTP rig (genus level). (DOCX)

S3 Table Impact of various factors on water chemistry from all Batch 1 samples (ANOSIM, Primer 6). (DOCX)

S4 Table Normality check of water chemistry data and Kruskal-Wallis analysis results. (DOCX)

S5 Table Pair-wise comparison of microbiome distance matrices across utilities (ANOSIM, permutation = 999). (DOCX)

S6 Table Impact of various factors on microbiome composition within each utility. (DOCX)

S7 Table Microbial Classes that contribute to over 80% similarity of samples within the same utility. (DOCX)

S8 Table Top 10 indicator taxa for each utility. (DOCX)

S9 Table Association of each variable with the microbiome across all Batch 1 samples. (DOCX)

S10 Table Summary of Utility characteristics. (DOCX)
