# Supplementary figures and images for: Impact of Water Chemistry, Pipe Material and Stagnation on the Building Plumbing Microbiome
Source: PLoS One. 2015 Oct 23;10(10):e0141087. doi: 10.1371/journal.pone.0141087 (PMC4619671; doi:10.1371/journal.pone.0141087)

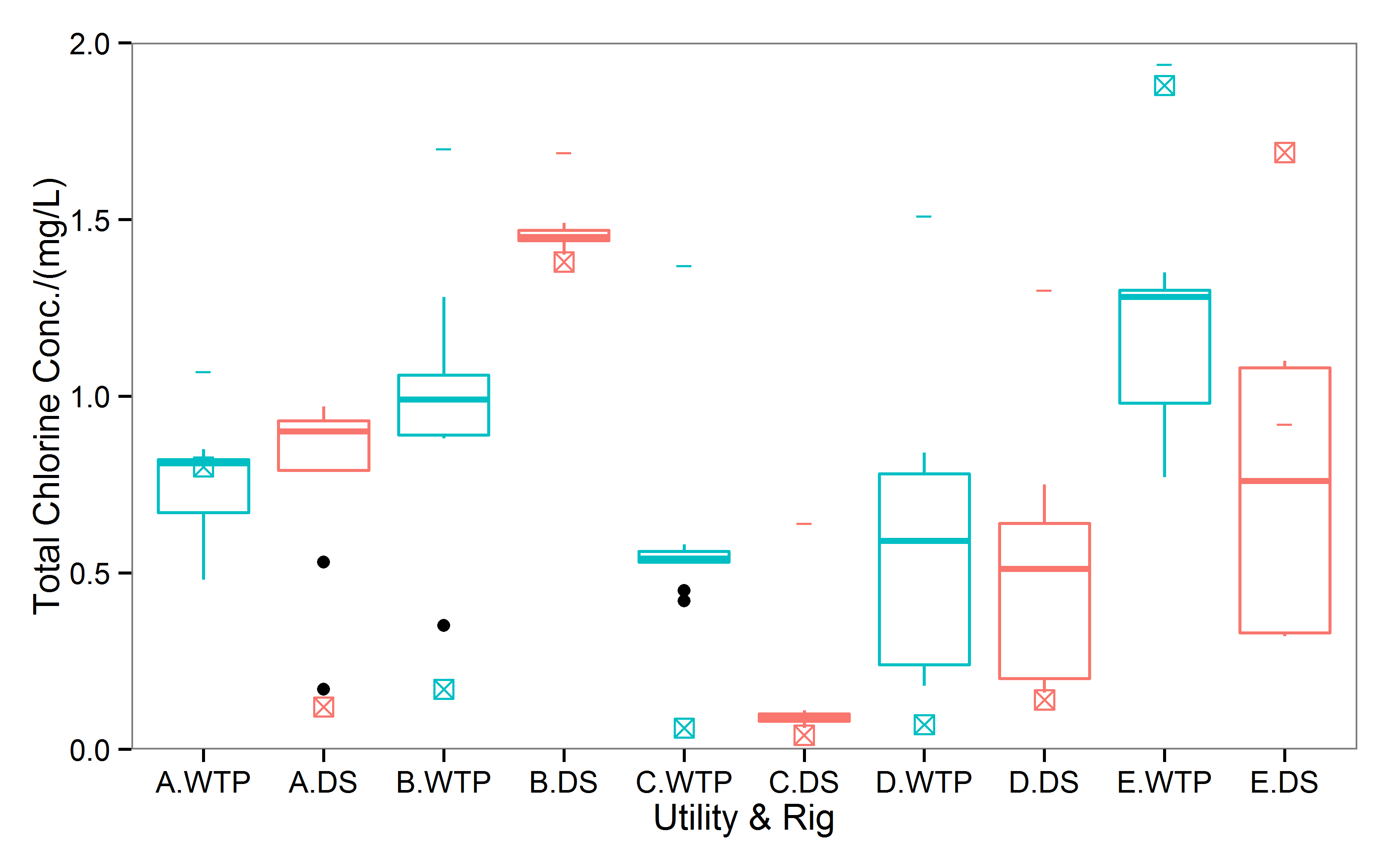

Supplement: S1 Fig — Each rig contains 9 pipe samples (triplicates of the three materials) following ~8hrs stagnation. Squares with cross inside are influent water samples, short lines represent average influent total chlorine concentration during 3-month prior to sampling event. Utilities A-D delivered chlorinated water, while Utility E delivered chloraminated water. The total chlorine concentration range was from 0.04–1.88 mg/L. (TIFF) [file pone.0141087.s001.tiff]

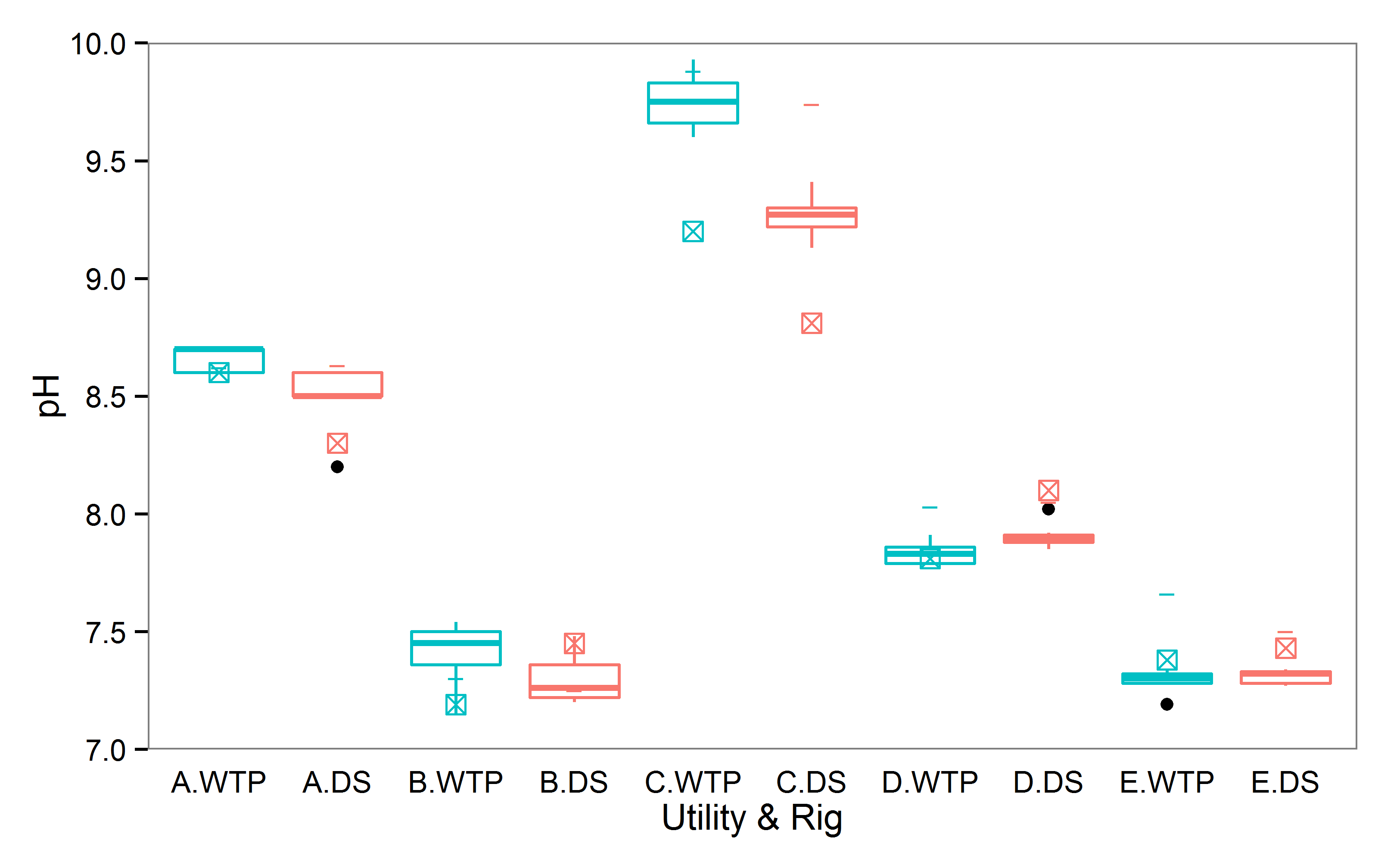

Supplement: S2 Fig — Each box contains 9 pipe samples from same rig (triplicates of the three materials) following ~8hrs stagnation. Squares with cross inside are influent water samples, while short lines are average influent pH values during 3-month prior to sampling. (TIFF) [file pone.0141087.s002.tiff]

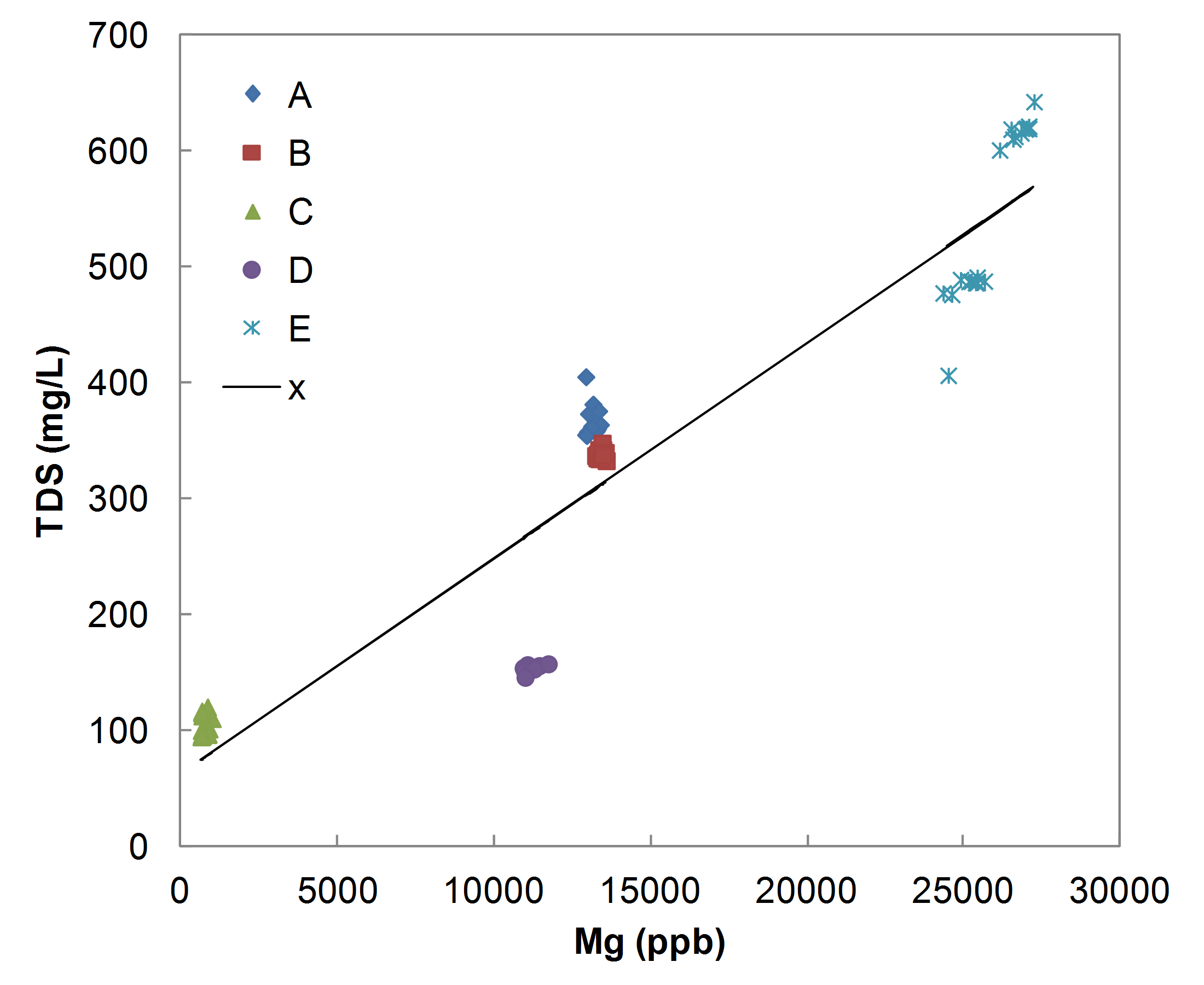

Supplement: S3 Fig — Total dissolved solids were calculated using water chemistry data, representing salinity of drinking water. (TIF) [file pone.0141087.s003.tif]
