# Supplementary material for: Impact of Water Chemistry, Pipe Material and Stagnation on the Building Plumbing Microbiome
Source: PLoS One. 2015 Oct 23;10(10):e0141087. doi: 10.1371/journal.pone.0141087 (PMC4619671; doi:10.1371/journal.pone.0141087)
Supplement: S1 Table — (DOCX) [file pone.0141087.s004.docx]

# S1 Table. Geographical distances among five utilities. City distance is estimated in miles based on shortest route by road.

|  | **A** | **B** | **C** | **D** | **E** |
| --- | --- | --- | --- | --- | --- |
| **A** | 0 | - | - | - | - |
| **B** | 12 | 0 | - | - | - |
| **C** | 844 | 823 | 0 | - | - |
| **D** | 373 | 380 | 1043 | 0 | - |
| **E** | 112 | 119 | 891 | 261 | 0 |
