# Supplementary material for: Impact of Water Chemistry, Pipe Material and Stagnation on the Building Plumbing Microbiome
Source: PLoS One. 2015 Oct 23;10(10):e0141087. doi: 10.1371/journal.pone.0141087 (PMC4619671; doi:10.1371/journal.pone.0141087)
Supplement: S2 Table — (DOCX) [file pone.0141087.s005.docx]

**S2 Table. Taxonomy composition of 3 field blanks from Utility A, WTP rig (genus level).** Sample names are Batch number when field blank was taken. Sequences number for each blank is in bracket. Minimum sequences per sample was 29238. Underlined are genera occurred in all three blanks.

| **Taxon** | **1**  **(943)** | **2**  **(1162)** | **3**  **(1212)** |
| --- | --- | --- | --- |
| k__Bacteria;p__Proteobacteria;c__Betaproteobacteria;o__Burkholderiales;f__Oxalobacteraceae;g__Ralstonia | 0.023 | 0.007 | 0.445 |
| k__Bacteria;p__Proteobacteria;c__Gammaproteobacteria;o__Pseudomonadales;f__Pseudomonadaceae;g__Pseudomonas | 0.015 | 0.076 | 0.002 |
| k__Bacteria;p__Proteobacteria;c__Gammaproteobacteria;o__Enterobacteriales;f__Enterobacteriaceae;g__ | 0.120 | 0.002 | 0.004 |
| k__Bacteria;p__Proteobacteria;c__Betaproteobacteria;o__Burkholderiales;f__Comamonadaceae;g__ | 0.006 | 0.067 | 0.002 |
| k__Bacteria;p__Proteobacteria;c__Alphaproteobacteria;o__Sphingomonadales;f__Sphingomonadaceae;g__Sphingomonas | 0.007 | 0.005 | 0.011 |
| k__Bacteria;p__Actinobacteria;c__Actinobacteria;o__Actinomycetales;f__Mycobacteriaceae;g__Mycobacterium | 0.004 | 0.010 | 0.004 |
| k__Bacteria;p__Proteobacteria;c__Alphaproteobacteria;o__Sphingomonadales;f__Erythrobacteraceae;g__ | 0.003 | 0.003 | 0.001 |
| k__Bacteria;p__Cyanobacteria;c__4C0d-2;o__MLE1-12;f__;g__ | 0.010 | 0.001 | 0.001 |
| k__Bacteria;p__Proteobacteria;c__Alphaproteobacteria;o__Sphingomonadales;f__Sphingomonadaceae;g__ | 0.003 | 0.003 | 0.001 |
| k__Bacteria;p__Proteobacteria;c__Alphaproteobacteria;o__Sphingomonadales;f__Sphingomonadaceae;g__Sphingopyxis | 0.003 | 0.001 | 0.002 |
| Unassigned;Other;Other;Other;Other;Other | 0.000 | 0.006 | 0.001 |
| k__Bacteria;p__Actinobacteria;c__Actinobacteria;o__Actinomycetales;f__Actinomycetaceae;g__Actinomyces | 0.000 | 0.000 | 0.073 |
| k__Bacteria;p__Actinobacteria;c__Actinobacteria;o__Actinomycetales;f__Corynebacteriaceae;g__Corynebacterium | 0.003 | 0.000 | 0.000 |
| k__Bacteria;p__Actinobacteria;c__Actinobacteria;o__Actinomycetales;f__Microbacteriaceae;g__ | 0.000 | 0.000 | 0.012 |
| k__Bacteria;p__Actinobacteria;c__Actinobacteria;o__Actinomycetales;f__Nocardiaceae;g__ | 0.009 | 0.000 | 0.007 |
| k__Bacteria;p__Actinobacteria;c__Actinobacteria;o__Actinomycetales;f__Nocardiaceae;g__Rhodococcus | 0.000 | 0.000 | 0.001 |
| k__Bacteria;p__Bacteroidetes;c__Sphingobacteriia;o__Sphingobacteriales;f__;g__ | 0.000 | 0.002 | 0.000 |
| k__Bacteria;p__Bacteroidetes;c__[Saprospirae];o__[Saprospirales];f__Chitinophagaceae;g__ | 0.003 | 0.000 | 0.002 |
| k__Bacteria;p__Bacteroidetes;c__[Saprospirae];o__[Saprospirales];f__Chitinophagaceae;g__Sediminibacterium | 0.104 | 0.000 | 0.000 |
| k__Bacteria;p__Chlamydiae;c__Chlamydiia;o__Chlamydiales;f__Rhabdochlamydiaceae;g__Candidatus Rhabdochlamydia | 0.000 | 0.001 | 0.001 |
| k__Bacteria;p__Chloroflexi;c__Chloroflexi;o__Herpetosiphonales;f__;g__ | 0.000 | 0.000 | 0.002 |
| k__Bacteria;p__Firmicutes;c__Bacilli;o__Bacillales;f__Alicyclobacillaceae;g__Alicyclobacillus | 0.000 | 0.253 | 0.000 |
| k__Bacteria;p__Firmicutes;c__Bacilli;o__Bacillales;f__Bacillaceae;Other | 0.000 | 0.001 | 0.000 |
| k__Bacteria;p__Firmicutes;c__Bacilli;o__Bacillales;f__Bacillaceae;g__Anoxybacillus | 0.000 | 0.181 | 0.000 |
| k__Bacteria;p__Firmicutes;c__Bacilli;o__Bacillales;f__Bacillaceae;g__Bacillus | 0.009 | 0.065 | 0.000 |
| k__Bacteria;p__Firmicutes;c__Bacilli;o__Bacillales;f__Paenibacillaceae;g__Paenibacillus | 0.000 | 0.050 | 0.000 |
| k__Bacteria;p__Firmicutes;c__Bacilli;o__Lactobacillales;f__Streptococcaceae;g__Streptococcus | 0.000 | 0.000 | 0.053 |
| k__Bacteria;p__Firmicutes;c__Clostridia;o__Clostridiales;f__Lachnospiraceae;g__Catonella | 0.000 | 0.000 | 0.041 |
| k__Bacteria;p__Fusobacteria;c__Fusobacteriia;o__Fusobacteriales;f__Leptotrichiaceae;g__Leptotrichia | 0.000 | 0.000 | 0.025 |
| k__Bacteria;p__Planctomycetes;c__BD7-11;o__;f__;g__ | 0.000 | 0.000 | 0.037 |
| k__Bacteria;p__Proteobacteria;c__Alphaproteobacteria;o__Caulobacterales;f__Caulobacteraceae;g__Phenylobacterium | 0.003 | 0.000 | 0.001 |
| k__Bacteria;p__Proteobacteria;c__Alphaproteobacteria;o__Rhizobiales;Other;Other | 0.000 | 0.001 | 0.000 |
| k__Bacteria;p__Proteobacteria;c__Alphaproteobacteria;o__Rhizobiales;f__;g__ | 0.000 | 0.182 | 0.001 |
| k__Bacteria;p__Proteobacteria;c__Alphaproteobacteria;o__Rhizobiales;f__Bradyrhizobiaceae;g__ | 0.000 | 0.002 | 0.002 |
| k__Bacteria;p__Proteobacteria;c__Alphaproteobacteria;o__Rhizobiales;f__Brucellaceae;g__Ochrobactrum | 0.000 | 0.001 | 0.000 |
| k__Bacteria;p__Proteobacteria;c__Alphaproteobacteria;o__Rhizobiales;f__Hyphomicrobiaceae;g__ | 0.000 | 0.001 | 0.000 |
| k__Bacteria;p__Proteobacteria;c__Alphaproteobacteria;o__Rhizobiales;f__Hyphomicrobiaceae;g__Hyphomicrobium | 0.000 | 0.002 | 0.002 |
| k__Bacteria;p__Proteobacteria;c__Alphaproteobacteria;o__Rhizobiales;f__Methylobacteriaceae;g__Methylobacterium | 0.002 | 0.000 | 0.001 |
| k__Bacteria;p__Proteobacteria;c__Alphaproteobacteria;o__Rhizobiales;f__Phyllobacteriaceae;g__Mesorhizobium | 0.028 | 0.000 | 0.000 |
| k__Bacteria;p__Proteobacteria;c__Alphaproteobacteria;o__Rhodospirillales;f__Acetobacteraceae;g__Acidocella | 0.000 | 0.000 | 0.031 |
| k__Bacteria;p__Proteobacteria;c__Alphaproteobacteria;o__Rhodospirillales;f__Rhodospirillaceae;g__ | 0.001 | 0.000 | 0.002 |
| k__Bacteria;p__Proteobacteria;c__Alphaproteobacteria;o__Sphingomonadales;f__Sphingomonadaceae;Other | 0.000 | 0.001 | 0.000 |
| k__Bacteria;p__Proteobacteria;c__Betaproteobacteria;Other;Other;Other | 0.000 | 0.000 | 0.002 |
| k__Bacteria;p__Proteobacteria;c__Betaproteobacteria;o__Burkholderiales;Other;Other | 0.000 | 0.000 | 0.001 |
| k__Bacteria;p__Proteobacteria;c__Betaproteobacteria;o__Burkholderiales;f__Burkholderiaceae;g__Burkholderia | 0.000 | 0.000 | 0.034 |
| k__Bacteria;p__Proteobacteria;c__Betaproteobacteria;o__Burkholderiales;f__Comamonadaceae;Other | 0.000 | 0.000 | 0.001 |
| k__Bacteria;p__Proteobacteria;c__Betaproteobacteria;o__Burkholderiales;f__Comamonadaceae;g__Acidovorax | 0.003 | 0.000 | 0.057 |
| k__Bacteria;p__Proteobacteria;c__Betaproteobacteria;o__Burkholderiales;f__Comamonadaceae;g__Comamonas | 0.004 | 0.000 | 0.000 |
| k__Bacteria;p__Proteobacteria;c__Betaproteobacteria;o__Burkholderiales;f__Comamonadaceae;g__Methylibium | 0.003 | 0.000 | 0.000 |
| k__Bacteria;p__Proteobacteria;c__Betaproteobacteria;o__Burkholderiales;f__Oxalobacteraceae;Other | 0.001 | 0.000 | 0.000 |
| k__Bacteria;p__Proteobacteria;c__Betaproteobacteria;o__Burkholderiales;f__Oxalobacteraceae;g__ | 0.000 | 0.000 | 0.004 |
| k__Bacteria;p__Proteobacteria;c__Betaproteobacteria;o__Methylophilales;f__Methylophilaceae;g__ | 0.000 | 0.000 | 0.002 |
| k__Bacteria;p__Proteobacteria;c__Betaproteobacteria;o__Neisseriales;f__Neisseriaceae;g__Neisseria | 0.000 | 0.000 | 0.051 |
| k__Bacteria;p__Proteobacteria;c__Betaproteobacteria;o__Procabacteriales;f__Procabacteriaceae;g__ | 0.000 | 0.000 | 0.025 |
| k__Bacteria;p__Proteobacteria;c__Deltaproteobacteria;o__Myxococcales;f__0319-6G20;g__ | 0.000 | 0.001 | 0.000 |
| k__Bacteria;p__Proteobacteria;c__Gammaproteobacteria;o__Pasteurellales;f__Pasteurellaceae;g__Aggregatibacter | 0.000 | 0.021 | 0.000 |
| k__Bacteria;p__Proteobacteria;c__Gammaproteobacteria;o__Pasteurellales;f__Pasteurellaceae;g__Haemophilus | 0.000 | 0.042 | 0.000 |
| k__Bacteria;p__Proteobacteria;c__Gammaproteobacteria;o__Pseudomonadales;f__Moraxellaceae;g__Acinetobacter | 0.259 | 0.008 | 0.000 |
| k__Bacteria;p__Proteobacteria;c__Gammaproteobacteria;o__Pseudomonadales;f__Pseudomonadaceae;Other | 0.028 | 0.000 | 0.000 |
| k__Bacteria;p__Proteobacteria;c__Gammaproteobacteria;o__Pseudomonadales;f__Pseudomonadaceae;g__ | 0.000 | 0.000 | 0.002 |
| k__Bacteria;p__Proteobacteria;c__Gammaproteobacteria;o__Xanthomonadales;f__Sinobacteraceae;g__ | 0.114 | 0.000 | 0.000 |
| k__Bacteria;p__Proteobacteria;c__Gammaproteobacteria;o__Xanthomonadales;f__Xanthomonadaceae;g__ | 0.093 | 0.000 | 0.055 |
| k__Bacteria;p__Proteobacteria;c__Gammaproteobacteria;o__Xanthomonadales;f__Xanthomonadaceae;g__Stenotrophomonas | 0.139 | 0.000 | 0.000 |
