# Supplementary material for: Impact of Water Chemistry, Pipe Material and Stagnation on the Building Plumbing Microbiome
Source: PLoS One. 2015 Oct 23;10(10):e0141087. doi: 10.1371/journal.pone.0141087 (PMC4619671; doi:10.1371/journal.pone.0141087)
Supplement: S3 Table — (DOCX) [file pone.0141087.s006.docx]

# S3 Table. Impact of various factors on water chemistry from all Batch 1 samples (ANOSIM, Primer 6). Euclidean distance matrix was constructed from normalized water chemistry data. “Strata” is based on sampling design, with permutation = 999.

| **Factor** | **Strata** | **Global R** | **P** |
| --- | --- | --- | --- |
| **Utility & Rig & Pipe** |  | 0.789 | 0.001 |
| **Utility.rig** |  | 0.720 | 0.001 |
| **Utility** |  | 0.713 | 0.001 |
| **Rig** | Utility | 0.332 | 0.001 |
| **Pipe Material** | Utility.rig | 0.448 | 0.001 |
| **Stagnation** | Utility.rig | 0.104 | ***0.182*** |
