# Supplementary material for: Impact of Water Chemistry, Pipe Material and Stagnation on the Building Plumbing Microbiome
Source: PLoS One. 2015 Oct 23;10(10):e0141087. doi: 10.1371/journal.pone.0141087 (PMC4619671; doi:10.1371/journal.pone.0141087)
Supplement: S4 Table — (DOCX) [file pone.0141087.s007.docx]

**S4 Table. Normality check of water chemistry data and Kruskal-Wallis analysis results.** Statistical significance were set at 0.05.

|  | **Shapiro-Wilk normality test** | | | **Kruskal-Wallis**  **(Utility)** | | **Kruskal-Wallis**  **(Pipe Material)** | | **Kruskal-Wallis**  **(Stagnation)** | |
| --- | --- | --- | --- | --- | --- | --- | --- | --- | --- |
|  | **W** | **P** | **Normality**  **(Y/N)** | **Chi-squared** | **P** | **Chi-squared** | **P** | **Chi-squared** | **P** |
| **Temperature** | 0.96 | 2.6e-03 | N | 28.0 | 1.3e-05 | ***5.21*** | ***0.16*** | ***2.48*** | ***0.12*** |
| **pH** | 0.88 | 1.9e-07 | N | 90.6 | < 2.2e-16 | ***0.0094*** | ***1 (0.9998)*** | ***0.001*** | ***0.97*** |
| **Total chlorine** | 0.96 | 2.8e-03 | N | 56.2 | 1.8e-11 | ***6.67*** | ***0.08*** | ***1.57*** | ***0.21*** |
| **Free chlorine^a^** | 0.92 | 1.4e-04 | N | 69.7 | 2.6e-14 | ***8.26*** | ***0.08*** | 4.90 | 0.03 |
| **Turbidity** | 0.25 | <2.2e-16 | N | 20.6 | 3.9e-04 | 8.79 | 0.03 | ***0.97*** | ***0.32*** |
| **Pb** | 0.36 | < 2.2e-16 | N | 19.4 | 6.5e-04 | 51.7 | 3.4e-11 | 10.1 | 0.002 |
| **Cu** | 0.66 | 8.8e-14 | N | 49.5 | 4.5e-10 | 39.0 | 1.7e-08 | 17.0 | 3.82e-05 |
| **Zn** | 0.65 | 3.4e-14 | N | ***7.63*** | ***0.10*** | 36.3 | 6.5e-08 | 3.84 | 0.05 |
| **Na** | 0.82 | 1.5e-09 | N | 95.1 | < 2.2e-16 | ***0.03*** | ***0.999*** | ***0.013*** | ***0.91*** |
| **Mg** | 0.85 | 9.3e-09 | N | 94.1 | < 2.2e-16 | ***0.21*** | ***0.98*** | ***0.10*** | ***0.75*** |
| **Al** | 0.87 | 6.6e-08 | N | 79.7 | < 2.2e-16 | ***1.38*** | ***0.71*** | ***0.57*** | ***0.45*** |
| **Si** | 0.73 | 3.1e-12 | N | 90.8 | < 2.2e-16 | ***0.08*** | ***0.99*** | ***0.05*** | ***0.83*** |
| **P** | 0.83 | 3.3e-09 | N | 92.0 | < 2.2e-16 | ***0.80*** | ***0.85*** | ***0.18*** | ***0.67*** |
| **NO_3_ as N** | 0.63 | 1.5e-14 | N | 47.8 | 1.1e-09 | ***1.39*** | ***0.71*** | ***1.20*** | ***0.27*** |
| **SO_4_** | 0.73 | 3.0e-12 | N | 79. 4 | 2.4e-16 | ***0.32*** | ***0.96*** | ***0.28*** | ***0.60*** |
| **TOC as C** | 0.88 | 1.6e-07 | N | 86.6 | < 2.2e-16 | ***1.17*** | ***0.76*** | ***0.57*** | ***0.45*** |
| **K** | 0.69 | 2.5e-13 | N | 94.3 | < 2.2e-16 | ***0.13*** | ***0.99*** | ***0.01*** | ***0.92*** |
| **Ca** | 0.81 | 6.7e-10 | N | 91.4 | < 2.2e-16 | ***0.27*** | ***0.97*** | ***0.11*** | ***0.74*** |
| **Fe** | 0.34 | < 2.2e-16 | N | 47.2 | 1.4e-09 | ***3.36*** | ***0.34*** | ***1.94*** | ***0.16*** |
| **F** | 0.93 | 5.7e-05 | N | 75.5 | 1.6e-15 | ***0.96*** | ***0.81*** | ***0.44*** | ***0.51*** |
| **Cl** | 0.84 | 6.0e-09 | N | 95.1 | < 2.2e-16 | ***0.29*** | ***0.96*** | ***0.11*** | ***0.74*** |

^a^ Free chlorine was compared among chlorinated utilities only (A-D): as free chlorine is not meaningful in chloraminated system.
