# Supplementary material for: Impact of Water Chemistry, Pipe Material and Stagnation on the Building Plumbing Microbiome
Source: PLoS One. 2015 Oct 23;10(10):e0141087. doi: 10.1371/journal.pone.0141087 (PMC4619671; doi:10.1371/journal.pone.0141087)
Supplement: S5 Table — (DOCX) [file pone.0141087.s008.docx]

# S5 Table. Pair-wise comparison of microbiome distance matrices across utilities (ANOSIM, permutation=999).

| **Utility Pair** | **Unweighted UniFrac** | | **Weighted UniFrac** | |
| --- | --- | --- | --- | --- |
|  | **Global R** | **P** | **Global R** | **P** |
| **A, E** | 0.886 | 0.001 | 0.394 | 0.001 |
| **A, B** | 0.948 | 0.001 | 0.249 | 0.001 |
| **A, C** | 0.997 | 0.001 | 0.883 | 0.001 |
| **A, D** | 0.955 | 0.001 | 0.451 | 0.001 |
| **E, B** | 0.93 | 0.001 | 0.399 | 0.001 |
| **E, C** | 0.997 | 0.001 | 0.757 | 0.001 |
| **E, D** | 0.85 | 0.001 | 0.398 | 0.001 |
| **B, C** | 1 | 0.001 | 0.869 | 0.001 |
| **B, D** | 0.696 | 0.001 | 0.497 | 0.001 |
| **C, D** | 0.999 | 0.001 | 0.822 | 0.001 |
