# Supplementary material for: Impact of Water Chemistry, Pipe Material and Stagnation on the Building Plumbing Microbiome
Source: PLoS One. 2015 Oct 23;10(10):e0141087. doi: 10.1371/journal.pone.0141087 (PMC4619671; doi:10.1371/journal.pone.0141087)
Supplement: S6 Table — (DOCX) [file pone.0141087.s009.docx]

# S6 Table. Impact of various factors on microbiome composition within each utility. Adonis test was applied using weighted UniFrac distance matrix. Impact size (R^2^) and significance (P) of four factors at each utility was reported. Data is in the form of R^2^ value (P value), with significance level set at 0.05.

| **Factor** | **Strata** | **A** | **B** | **C** | **D** | **E** |
| --- | --- | --- | --- | --- | --- | --- |
| **Rig** |  | 0.245 (0.001) | 0.176 (0.001) | 0.379 (0.001) | 0.144 (0.001) | 0.176 (0.001) |
| **Pipe material** | Rig | 0.105 (0.002) | 0.097 (0.012) | 0.124 (0.001) | 0.211 (0.001) | 0.324 (0.001) |
| **Stagnation** | Rig | 0.038 (0.023) | 0.035 (***0.054***) | 0.092 (0.001) | 0.085 (0.001) | 0.128 (0.001) |
| **Batch** | Rig.Pipe | 0.039 (0.011) | 0.041 (0.016) | 0.028 (0.010) | 0.026 (0.011) | 0.004 (***0.818***) |
