# Supplementary material for: Impact of Water Chemistry, Pipe Material and Stagnation on the Building Plumbing Microbiome
Source: PLoS One. 2015 Oct 23;10(10):e0141087. doi: 10.1371/journal.pone.0141087 (PMC4619671; doi:10.1371/journal.pone.0141087)
Supplement: S7 Table — (DOCX) [file pone.0141087.s010.docx]

# S7 Table. Microbial classes that contribute to over 80% similarity of samples within the same utility.

| **Utility** | **Class** | **Avg. Abund.** | **Contrib%** | **Cum.%** |
| --- | --- | --- | --- | --- |
| **A (69% average similarity)** | *Alphaproteobacteria* | 0.41 | 48 | 48 |
|  | *Actinobacteria* | 0.31 | 32 | 80 |
|  | *4C0d-2* | 0.08 | 8.2 | 88 |
| **B (76% average similarity)** | *Alphaproteobacteria* | 0.49 | 55 | 55 |
|  | *Actinobacteria* | 0.28 | 28 | 83 |
| **C (77% average similarity)** | *Alphaproteobacteria* | 0.72 | 80 | 80 |
|  | *Betaproteobacteria* | 0.18 | 16 | 95 |
| **D (66% average similarity)** | *Alphaproteobacteria* | 0.32 | 41 | 41 |
|  | *Betaproteobacteria* | 0.19 | 16 | 56 |
|  | *Actinobacteria* | 0.15 | 15 | 72 |
|  | *Gammaproteobacteria* | 0.15 | 12 | 84 |
| **E (69% average similarity)** | *Alphaproteobacteria* | 0.44 | 52 | 52 |
|  | *Betaproteobacteria* | 0.22 | 21 | 74 |
|  | *Actinobacteria* | 0.15 | 14 | 88 |
