# Supplementary material for: Impact of Water Chemistry, Pipe Material and Stagnation on the Building Plumbing Microbiome
Source: PLoS One. 2015 Oct 23;10(10):e0141087. doi: 10.1371/journal.pone.0141087 (PMC4619671; doi:10.1371/journal.pone.0141087)
Supplement: S8 Table — (DOCX) [file pone.0141087.s011.docx]

**S8 Table. Top 10 indicator taxa for each utility**. Indicator value was calculated using package {indicspecies} in R.

| **Utility** | **Taxonomy** | **Indicator value** | **P** | **Avg. RA^a^** |
| --- | --- | --- | --- | --- |
| **A** | k__Bacteria;p__Firmicutes;c__Bacilli;o__Bacillales;f__Staphylococcaceae;g__Staphylococcus | 0.863 | 0.0002 | 9.23E-03 |
|  | k__Bacteria;p__Planctomycetes;c__Phycisphaerae;o__Phycisphaerales;f__;g__ | 0.844 | 0.0002 | 3.53E-03 |
|  | k__Bacteria;p__Proteobacteria;c__Alphaproteobacteria;o__Sphingomonadales;f__;g__ | 0.812 | 0.0002 | 3.08E-02 |
|  | k__Bacteria;p__Proteobacteria;c__Alphaproteobacteria;o__Sphingomonadales;f__Sphingomonadaceae;g__Novosphingobium | 0.804 | 0.0002 | 5.93E-03 |
|  | k__Bacteria;p__Firmicutes;c__Bacilli;o__Bacillales;f__Alicyclobacillaceae;g__Alicyclobacillus | 0.769 | 0.0002 | 8.43E-03 |
|  | k__Bacteria;p__Proteobacteria;c__Gammaproteobacteria;o__Xanthomonadales;f__Xanthomonadaceae;g__ | 0.746 | 0.0002 | 3.06E-03 |
|  | k__Bacteria;p__Proteobacteria;c__Gammaproteobacteria;o__Pseudomonadales;f__Pseudomonadaceae;g__Pseudomonas | 0.711 | 0.0006 | 1.54E-02 |
|  | k__Bacteria;p__Proteobacteria;c__Alphaproteobacteria;o__Rhizobiales;f__Rhizobiaceae;g__Agrobacterium | 0.704 | 0.0004 | 5.89E-03 |
|  | k__Bacteria;p__Proteobacteria;c__Betaproteobacteria;o__Burkholderiales;f__Comamonadaceae;g__Acidovorax | 0.675 | 0.0002 | 6.39E-02 |
|  | k__Bacteria;p__Proteobacteria;c__Alphaproteobacteria;o__Rhodobacterales;f__Hyphomonadaceae;g__ | 0.653 | 0.0002 | 1.64E-02 |
| **B** | k__Bacteria;p__Proteobacteria;c__Betaproteobacteria;o__Burkholderiales;f__Comamonadaceae;g__Hydrogenophaga | 0.787 | 0.0002 | 3.33E-02 |
|  | k__Bacteria;p__Proteobacteria;c__Alphaproteobacteria;o__Caulobacterales;f__Caulobacteraceae;g__Mycoplana | 0.636 | 0.0006 | 2.86E-02 |
|  | k__Bacteria;p__Proteobacteria;c__Alphaproteobacteria;o__Caulobacterales;f__Caulobacteraceae;g__Caulobacter | 0.585 | 0.0096 | 3.41E-03 |
|  | k__Bacteria;p__Proteobacteria;c__Alphaproteobacteria;o__Sphingomonadales;f__Erythrobacteraceae;g__Erythrobacter | 0.528 | 0.0002 | 6.02E-05 |
|  | k__Bacteria;p__Cyanobacteria;c__4C0d-2;o__MLE1-12;f__;g__ | 0.528 | 0.0016 | 1.12E-01 |
|  | k__Bacteria;p__Firmicutes;c__Erysipelotrichi;o__Erysipelotrichales;f__Erysipelotrichaceae;g__PSB-M-3 | 0.34 | 0.0002 | 3.55E-05 |
|  | k__Bacteria;p__Proteobacteria;c__Gammaproteobacteria;o__Pseudomonadales;Other;Other | 0.313 | 0.0002 | 8.87E-06 |
|  | k__Bacteria;p__Firmicutes;c__Clostridia;o__OPB54;f__;g__ | 0.265 | 0.005 | 3.36E-05 |
|  | k__Bacteria;p__Proteobacteria;c__Gammaproteobacteria;o__Alteromonadales;f__Alteromonadaceae;g__Cellvibrio | 0.262 | 0.0038 | 6.97E-06 |
|  | k__Bacteria;p__Gemmatimonadetes;c__Gemmatimonadetes;o__Ellin5290;f__;g__ | 0.236 | 0.0078 | 2.53E-05 |
| **C** | k__Bacteria;p__Verrucomicrobia;c__[Methylacidiphilae];o__Methylacidiphilales;f__LD19;g__ | 0.991 | 0.0002 | 8.44E-03 |
|  | k__Bacteria;p__Proteobacteria;c__Gammaproteobacteria;o__Methylococcales;f__Methylococcaceae;g__Methylocaldum | 0.991 | 0.0002 | 5.08E-03 |
|  | k__Bacteria;p__Proteobacteria;c__Gammaproteobacteria;o__Legionellales;f__Legionellaceae;g__ | 0.981 | 0.0002 | 6.53E-03 |
|  | k__Bacteria;p__Proteobacteria;c__Alphaproteobacteria;o__Rhizobiales;f__Hyphomicrobiaceae;g__ | 0.971 | 0.0002 | 2.92E-03 |
|  | k__Bacteria;p__Proteobacteria;c__Betaproteobacteria;o__Methylophilales;f__Methylophilaceae;g__ | 0.952 | 0.0002 | 2.74E-02 |
|  | k__Bacteria;p__TM6;c__;o__;f__;g__ | 0.939 | 0.0002 | 1.47E-03 |
|  | k__Bacteria;p__Proteobacteria;c__Alphaproteobacteria;o__Rhizobiales;f__Hyphomicrobiaceae;g__Hyphomicrobium | 0.91 | 0.0002 | 9.36E-02 |
|  | k__Bacteria;p__Proteobacteria;c__Betaproteobacteria;o__Burkholderiales;f__Oxalobacteraceae;Other | 0.907 | 0.0002 | 8.82E-04 |
|  | k__Bacteria;p__Proteobacteria;c__Betaproteobacteria;o__Methylophilales;f__Methylophilaceae;Other | 0.903 | 0.0002 | 5.61E-04 |
|  | k__Bacteria;p__Proteobacteria;c__Alphaproteobacteria;o__Rhizobiales;f__Hyphomicrobiaceae;Other | 0.870 | 0.0002 | 2.82E-04 |
|  | k__Bacteria;p__Proteobacteria;c__Alphaproteobacteria;o__Rhodospirillales;f__Acetobacteraceae;g__ | 0.857 | 0.0002 | 4.10E-03 |
|  | k__Bacteria;p__Proteobacteria;c__Betaproteobacteria;o__Burkholderiales;f__Comamonadaceae;g__Limnobacter | 0.852 | 0.0002 | 5.94E-03 |
|  | k__Bacteria;p__Proteobacteria;c__Gammaproteobacteria;o__Legionellales;f__Legionellaceae;Other | 0.843 | 0.0002 | 2.92E-04 |
|  | k__Bacteria;p__Proteobacteria;c__Alphaproteobacteria;o__;f__;g__ | 0.839 | 0.0002 | 1.85E-02 |
|  | k__Bacteria;p__Proteobacteria;c__Betaproteobacteria;o__Burkholderiales;f__Burkholderiaceae;g__Burkholderia | 0.834 | 0.0002 | 2.52E-03 |
|  | k__Bacteria;p__Proteobacteria;c__Betaproteobacteria;o__Burkholderiales;f__Oxalobacteraceae;g__Ralstonia | 0.833 | 0.0002 | 1.18E-01 |
|  | k__Bacteria;p__Proteobacteria;c__Gammaproteobacteria;o__Legionellales;f__Legionellaceae;g__Legionella | 0.828 | 0.0002 | 3.05E-04 |
|  | k__Bacteria;p__Chlamydiae;c__Chlamydiia;o__Chlamydiales;f__;g__ | 0.824 | 0.0002 | 5.08E-04 |
|  | k__Bacteria;p__Proteobacteria;c__Alphaproteobacteria;o__Sphingomonadales;f__Sphingomonadaceae;g__Blastomonas | 0.811 | 0.0002 | 4.43E-03 |
|  | k__Bacteria;p__Proteobacteria;c__Betaproteobacteria;o__Burkholderiales;Other;Other | 0.809 | 0.0002 | 3.95E-04 |
|  | k__Bacteria;p__Proteobacteria;c__Betaproteobacteria;o__Burkholderiales;f__;g__ | 0.796 | 0.0002 | 4.81E-04 |
| D | k__Bacteria;p__Proteobacteria;c__Gammaproteobacteria;o__Xanthomonadales;f__Sinobacteraceae;g__Nevskia | 0.929 | 0.0002 | 1.36E-01 |
|  | k__Bacteria;p__Bacteroidetes;c__Cytophagia;o__Cytophagales;f__Cytophagaceae;g__ | 0.889 | 0.0002 | 1.43E-02 |
|  | k__Bacteria;p__Proteobacteria;c__Betaproteobacteria;o__Burkholderiales;f__Comamonadaceae;g__Polaromonas | 0.885 | 0.0002 | 3.47E-04 |
|  | k__Bacteria;p__Proteobacteria;c__Alphaproteobacteria;o__Sphingomonadales;f__Sphingomonadaceae;g__Sphingobium | 0.799 | 0.0002 | 1.40E-02 |
|  | k__Bacteria;p__Proteobacteria;c__Betaproteobacteria;o__Burkholderiales;f__Comamonadaceae;g__Methylibium | 0.744 | 0.0002 | 1.65E-02 |
|  | k__Bacteria;p__Proteobacteria;c__Alphaproteobacteria;o__Rhodospirillales;f__Acetobacteraceae;g__Roseococcus | 0.713 | 0.0002 | 1.29E-03 |
|  | k__Bacteria;p__Acidobacteria;c__Solibacteres;o__Solibacterales;f__[Bryobacteraceae];g__ | 0.713 | 0.0002 | 1.27E-03 |
|  | k__Bacteria;p__Bacteroidetes;c__[Saprospirae];o__[Saprospirales];f__Chitinophagaceae;g__ | 0.694 | 0.0002 | 1.33E-02 |
|  | k__Bacteria;p__Proteobacteria;c__Alphaproteobacteria;o__Rhodobacterales;f__Rhodobacteraceae;g__ | 0.675 | 0.0076 | 7.78E-04 |
|  | k__Bacteria;p__Bacteroidetes;c__Flavobacteriia;o__Flavobacteriales;f__Cryomorphaceae;g__Fluviicola | 0.670 | 0.0002 | 8.45E-04 |
| E | k__Bacteria;p__Proteobacteria;c__Betaproteobacteria;o__Nitrosomonadales;f__Nitrosomonadaceae;g__ | 0.998 | 0.0002 | 1.27E-02 |
|  | k__Bacteria;p__Planctomycetes;c__Planctomycetia;o__Planctomycetales;f__Planctomycetaceae;g__Planctomyces | 0.976 | 0.0002 | 8.90E-03 |
|  | k__Bacteria;p__Proteobacteria;c__Alphaproteobacteria;o__Rhizobiales;f__Rhizobiaceae;g__ | 0.969 | 0.0002 | 3.88E-03 |
|  | k__Bacteria;p__Proteobacteria;c__Alphaproteobacteria;o__Rhizobiales;f__Rhizobiaceae;Other | 0.942 | 0.0002 | 2.82E-03 |
|  | k__Bacteria;p__Planctomycetes;c__Planctomycetia;o__Pirellulales;f__Pirellulaceae;g__Pirellula | 0.891 | 0.0002 | 4.61E-04 |
|  | k__Bacteria;p__Bacteroidetes;c__Sphingobacteriia;o__Sphingobacteriales;f__;g__ | 0.830 | 0.0002 | 7.99E-03 |
|  | k__Bacteria;p__Nitrospirae;c__Nitrospira;o__Nitrospirales;f__Nitrospiraceae;g__Nitrospira | 0.796 | 0.0002 | 1.74E-03 |
|  | k__Bacteria;p__Proteobacteria;c__Alphaproteobacteria;o__Rhizobiales;f__Methylobacteriaceae;g__Methylobacterium | 0.776 | 0.0002 | 6.78E-02 |
|  | k__Bacteria;p__Proteobacteria;c__Betaproteobacteria;o__Rhodocyclales;f__Rhodocyclaceae;g__ | 0.773 | 0.0002 | 1.53E-03 |
|  | k__Bacteria;p__Proteobacteria;c__Alphaproteobacteria;o__Sphingomonadales;f__Sphingomonadaceae;g__Sphingopyxis | 0.748 | 0.0002 | 5.74E-02 |

^a^ Average RA is calculated based on average number of RA within same utility samples using taxonomy table at genus level (rarefied table). Rank is within each utility’s samples and omitted “0” ones.
