# Supplementary material for: Impact of Water Chemistry, Pipe Material and Stagnation on the Building Plumbing Microbiome
Source: PLoS One. 2015 Oct 23;10(10):e0141087. doi: 10.1371/journal.pone.0141087 (PMC4619671; doi:10.1371/journal.pone.0141087)
Supplement: S9 Table — (DOCX) [file pone.0141087.s012.docx]

# S9 Table. Association of each variable with the microbiome across all Batch 1 samples. (adonis, permutations = 999, alpha = 0.05, {vegan}, R)

| **Parameter** | **Unweighted UniFrac** | | **Weighted UniFrac** | |
| --- | --- | --- | --- | --- |
|  | **R^2^** | **Pr(>F)** | **R^2^** | **Pr(>F)** |
| **Mg** (ppb) | 0.065 | 0.001 | 0.132 | 0.001 |
| **pH** | 0.075 | 0.001 | 0.131 | 0.001 |
| **F** (mg/L) | 0.051 | 0.001 | 0.118 | 0.001 |
| **P** (ppb) | 0.066 | 0.001 | 0.114 | 0.001 |
| **SO_4_** (mg/L) | 0.048 | 0.001 | 0.108 | 0.001 |
| **Ca** (ppb) | 0.056 | 0.001 | 0.104 | 0.001 |
| **Free chlorine** (mg/L) | 0.037 | 0.001 | 0.082 | 0.001 |
| **Na** (ppb) | 0.045 | 0.001 | 0.082 | 0.001 |
| **Al** (ppb) | 0.047 | 0.001 | 0.078 | 0.001 |
| **K** (ppb) | 0.037 | 0.001 | 0.069 | 0.001 |
| **Total chlorine** (mg/L) | 0.036 | 0.001 | 0.069 | 0.001 |
| **Cl** (mg/L) | 0.043 | 0.001 | 0.069 | 0.002 |
| **Si** (ppb) | 0.041 | 0.001 | 0.063 | 0.016 |
| **Temperature** (F) | 0.030 | 0.003 | 0.061 | 0.001 |
| **TOC as C** (mg/L) | 0.031 | 0.004 | 0.053 | 0.025 |
| **Cu** (ppb) | 0.028 | 0.001 | 0.039 | 0.022 |
| **Zn** (ppb) | 0.016 | 0.004 | 0.023 | 0.033 |
| **Fe** (ppb) | 0.016 | 0.011 | 0.020 | 0.047 |
| **Pb** (ppb) | 0.016 | 0.008 | 0.017 | ***0.102*** |
| **NO_3_ as N** (mg/L) | 0.015 | 0.011 | 0.011 | ***0.325*** |
| **Turbidity** (NTU) | 0.011 | ***0.143*** | 0.007 | ***0.632*** |
