# Supplementary material for: Impact of Water Chemistry, Pipe Material and Stagnation on the Building Plumbing Microbiome
Source: PLoS One. 2015 Oct 23;10(10):e0141087. doi: 10.1371/journal.pone.0141087 (PMC4619671; doi:10.1371/journal.pone.0141087)
Supplement: S10 Table — (DOCX) [file pone.0141087.s013.docx]

# S10 Table. Summary of Utility characteristics.

| **Utility** | **Sampling Time** | **Treatment Process** | **Disinfectant Type** |
| --- | --- | --- | --- |
| **A** | Nov. 27-29^th^, 2012 | Conventional treatment plus GAC contactors, UV disinfection as second barrier, chlorine disinfectant, and corrosion inhibitor. | Chlorine |
| **B** | Dec. 12-13^th^, 2012 | Conventional treatment, chlorine disinfectant and corrosion inhibitor. | Chlorine |
| **C** | Nov. 27-29^th^, 2012 | Conventional treatment with chlorine disinfection, lime and CO_2_ added for water stability, no corrosion inhibitor. | Chlorine |
| **D** | Dec. 17-18^th^, 2012 | Conventional treatment plus ultra-filtration membranes, chlorine disinfection and corrosion inhibitor. | Chlorine |
| **E** | Jan. 13-16^th^, 2013 | Conventional treatment with UV disinfection and chloramines disinfection, no corrosion inhibitor. | Chloramine |
